# The iron/heme regulated genes of *Haemophilus influenzae*: comparative transcriptional profiling as a tool to define the species core modulon

**DOI:** 10.1186/1471-2164-10-6

**Published:** 2009-01-07

**Authors:** Paul W Whitby, Thomas W Seale, Timothy M VanWagoner, Daniel J Morton, Terrence L Stull

**Affiliations:** 1 Department of Pediatrics, University of Oklahoma Health Sciences Center, Oklahoma City, OK, 73104, USA; 2 Department of Microbiology and Immunology, University of Oklahoma Health Sciences Center, Oklahoma City, OK, 73104, USA; 3 Department of Biology, Oklahoma Christian University, Oklahoma City, OK 73136, USA

## Abstract

**Background:**

*Haemophilus influenzae *requires heme for aerobic growth and possesses multiple mechanisms to obtain this essential nutrient. Although an understanding of the heme acquisition mechanisms of *H. influenzae *is emerging, significant gaps in our knowledge remain. Unresolved issues include the identities of all genes exhibiting altered transcription in response to iron and heme availability, the fraction of such genes functioning in iron/heme acquisition, and the heterogeneity of this gene set among clinical isolates. Previously we utilized *H. influenzae *strain Rd KW20 to demonstrate the utility of transcriptional profiling in defining the genes exhibiting altered transcription in response to environmental iron and heme levels. The current study expands upon those observations by determining the iron/heme modulons of two clinical isolates, the type b isolate 10810 and the nontypeable isolate R2866. These data are used to begin to define the core iron/heme modulon of the species.

**Results:**

Microarray studies were performed to compare gene expression on transition from iron/heme-restricted to iron/heme-replete conditions for each isolate. Of 1820 ORFs on the array corresponding to R2866 genes, 363 were significantly differentially expressed: 233 were maximally transcribed under iron/heme-replete conditions and 130 under iron/heme-restricted conditions. Of the 1883 ORFs representing genes of strain 10810, 353 were significantly differentially transcribed: 150 were preferentially transcribed under iron/heme-replete conditions and 203 under iron/heme-restricted conditions. Comparison of the data sets indicated that 163 genes exhibited similar regulation in both isolates and that 74 of these exhibited similar patterns of regulation in Rd KW20. These comprise the putative core iron/heme modulon.

**Conclusion:**

This study provides evidence for a conserved core of *H. influenzae *genes the transcription of which is altered by the availability of iron and/or heme in the growth environment. Elucidation of this modulon provides a means to identify genes with unrecognized roles in iron/heme acquisition or homeostasis, unanticipated responsiveness to environmental levels of the micronutrients or potential roles in virulence. Defining these core genes is also of potential importance in identifying targets for therapeutic and vaccine designs since products of these genes are likely to be preferentially expressed during growth in iron/heme restricted sites of the human body.

## Background

*Haemophilus influenzae *is a fastidious, Gram-negative, facultatively anaerobic, opportunistic pathogen, and is found only in man where it is a common commensal in the nasopharynx [1,2]. *H*.*influenzae *is a frequent cause of both invasive and non-invasive diseases including epiglottitis, pneumonia, bacteremia, meningitis, respiratory infections and otitis media (OM) [1,3-5]. OM due to *H. influenzae *is principally caused by nontypeable *H. influenzae *(NTHi) strains [6], while invasive disease can be caused by either encapsulated or unencapsulated strains. Of the six biochemically and antigenically distinct capsular types (a-f), *H. influenzae *serotype b (Hib) strains accounted for the vast majority of bacteremia and meningitis cases prior to the introduction of vaccines based on the type b capsule [5]. While such vaccines have nearly eradicated meningitis caused by type b strains in the developed world [5], they lack effectiveness against other capsular types [7,8] and against nontypeable strains [9] which remain a significant public health issue.

*H. influenzae *has an absolute aerobic growth requirement for heme; since most strains of *H. influenzae *possess the enzyme ferrochelatase, the requirement is specifically for the immediate heme precursor protoporphyrin IX (PPIX) [10]. In the human host, heme is intracellular in the form of globins or other heme containing proteins, and thus unavailable to invading microorganisms. However, *H. influenzae *is able to use many potential host derived hemoproteins to satisfy its heme requirements, including hemoglobin, hemoglobin-haptoglobin, myoglobin-haptoglobin, heme-hemopexin, heme-albumin and catalase [11-15]. In addition *H. influenzae *can grow when supplied with PPIX in the presence of an iron source such as ferri-transferrin [13,16,17].

**Figure 1:**
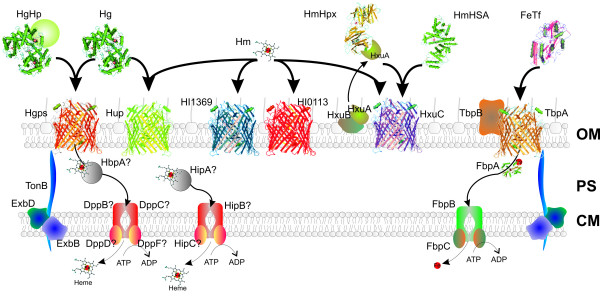
**A schematic of our current understanding of the heme and iron acquisition systems of *H. influenzae***. Transport across the outer membrane (OM) of iron and heme from various sources is outlined. Heme from hemoglobin (Hg) can be acquired through the TonB-dependent Hgp and Hup proteins [69,70]. Heme from hemoglobin-haptoglobin complex (HgHp) is acquired through the Hgps [69]. Heme from heme-hemopexin (HmHpx) is acquired through HxuC, and is dependent on the proteins HxuB and HxuA [71,72]. Acquisition of heme from heme-human serum albumin (HmHSA) is mediated by HxuC independently of HxuB and HxuA [72]. The role of the TonB-dependent receptor proteins HI1369 and HI0113 has not been experimentally determined although we postulate that along with HxuC and Hup they may act as redundant mediators for acquisition of free heme. Acquisition of transferrin-bound iron requires the TonB-dependent protein TbpA and the accessory protein TbpB [17]. Transport systems for other iron sources and PPIX across the OM have not been determined. Transport across the cytoplasmic membrane is less well characterized. The *H. influenzae *strains appear to have redundant heme and iron ABC transport systems and multiple candidates for these systems have been discovered. HbpA is a periplasmic heme transporter and may deliver heme to the DppBCDF membrane transporter [55]. The Hib *hip *locus has also been shown to be involved in heme transport [46]. Iron is transported into the cell via HitABC (FbpABC). Mutations of *hpbA *or *hitABC *do not abolish utilization of heme and iron respectively, indicating additional cytoplasmic transport mechanisms. Lipoprotein *e*(P4) has also been shown to involved in heme acquisition although it is not included in this figure since little is known about its mode of action [21].

The mechanisms mediating utilization of these various heme and iron sources by *H. influenzae *are complex and highly redundant. Our current understanding of the *H. influenzae *heme and iron acquisition systems is summarized in Figure 1 and has recently been reviewed by Morton and Stull [18]. Several of the heme acquisition associated proteins have been shown to be involved in virulence in animal models of both invasive and non-invasive *H. influenzae *disease [19-22]. Significant gaps remain in our understanding of *H. influenzae *iron/heme acquisition, and the elucidation of the core iron and/or heme (FeHm) modulon of *H. influenzae *will potentially provide additional valuable insights into the mechanisms involved in the utilization of these essential nutrients.

Since iron and heme sources are sequestered within the human host [23], it is likely that the host microenvironment will result in the increased expression of *H. influenzae *proteins encoded by genes preferentially transcribed during growth in FeHm-restricted media in vitro. Evidence for this is provided by our previous report of transcription of several *H. influenzae *FeHm-acquisition associated genes in middle ear effusions from patients with OM [24]. Identification of the FeHm regulated genes in *H. influenzae *would thus potentially further elucidate the FeHm acquisition pathways as well as identify previously unknown virulence determinants.

We previously reported a microarray based study to identify the FeHm-responsive genes of the sequenced *H. influenzae *isolate Rd KW20 [25]. Both this previous study, as well as the current one, examine the effect of iron and heme together since it is not currently technically possible to separate the effects of these two molecules on transcription [25]. The previous study identified in excess of 80 genes that were significantly upregulated under FeHm restricted conditions. Many of these genes have been previously studied and been associated with FeHm uptake, utilization and/or homeostasis. Other identified genes had defined roles in metabolism while several additional genes were uncharacterized. However, since the type d parent of Rd KW20 was originally isolated over 60 years ago [26,27] and has undergone extensive passaging on laboratory media, this isolate is unlikely to be representative of clinically important strains and may even be altered in its regulation of gene expression. Indeed, since Rd KW20 is derived from a type d strain and was selected as a rough derivative following loss of the capsule, at least one major genetic event has occurred since primary isolation [26,27]. Thus, it is important to examine the FeHm modulons of more representative clinical isolates. Two invasive isolates were selected for further microarray analysis, the serotype b isolate 10810 was from a patient with meningitis [28] and the NTHi strain R2866 isolated from the blood of an immunocompetent child with clinical signs of meningitis subsequent to acute OM [29]. The primary goal of these studies is to begin to define the core set of genes preferentially transcribed under FeHm limitation in clinically important isolates of *H. influenzae *and thereby identify potential therapeutic and vaccine targets expressed broadly across the species.

## Results and Discussion

### Q-PCR Analysis: Derepression of transcription in FeHm restricted media

**Figure 2:**
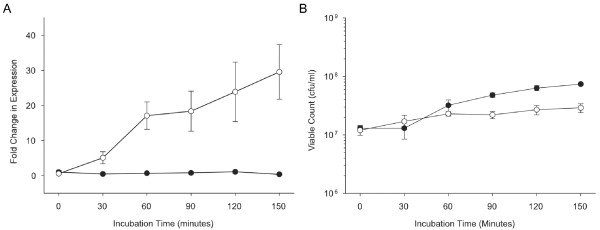
**Derepression kinetics defining the window of transcriptional regulation for the FeHm-regulated gene *tbp1 *in strain Hib 10810**. (A) Fold change in expression of *tbp1 *in Hib strain 10810 over 150 minutes of growth in either FeHm-replete (closed circles) or FeHm-restricted (open circles) media. (B) Viable counts of strain Hib 10810 over 150 minutes of growth in either FeHm-replete (closed circles) or FeHm-restricted (open circles) media.

Since our goal was the identification of genes that are preferentially transcribed under conditions of FeHm limitation, it was necessary to first describe the kinetics of transcription of genes known to be FeHm repressible in the two clinical isolates. Genes encoding the periplasmic iron-binding protein HitA, the transferrin-binding protein TbpA and the heme/hemopexin uptake protein HxuC were utilized for this purpose. Transcript levels were determined by Q-PCR following introduction of bacteria into either FeHm-replete or FeHm-restricted media for each experimental isolate (Hib 10810 or NTHi R2866) essentially as described previously for strain Rd KW20 [25]. Figure 2A shows transcription of *tbp1 *in the Hib strain 10810 over a 150 minute period following introduction of cells into the test media. The transcript levels of *tbp1 *increased to a plateau level within 90 minutes of growth in the FeHm-restricted media (BHI containing 150 μM deferroxamine) but remained at the baseline level during growth in FeHm-replete media. Thus, growth in FeHm-deplete media promoted transcription of this gene. To ensure that the lack of increase in transcripts in the FeHm-replete media was not due to cell death, viable counts were taken at each time point. The viable count data demonstrated that strain 10810 retained viability for the duration of the experiment in both FeHm-replete and -restricted cultures (Figure 2B). Transcript levels of *hitA *and *hxuC *exhibited profiles similar to that observed for the *tbp1 *transcripts (data not shown). Transcription of these three genes in strain R2866 was essentially the same (data not shown). Replicate experiments conducted on different occasions established that the time course of change in transcript levels, magnitude of transcriptional changes and growth profiles were consistent and reproducible for both Hib 10810 and NTHi R2866.

### Q-PCR Analysis: Repression of transcription in FeHm supplemented media

**Figure 3:**
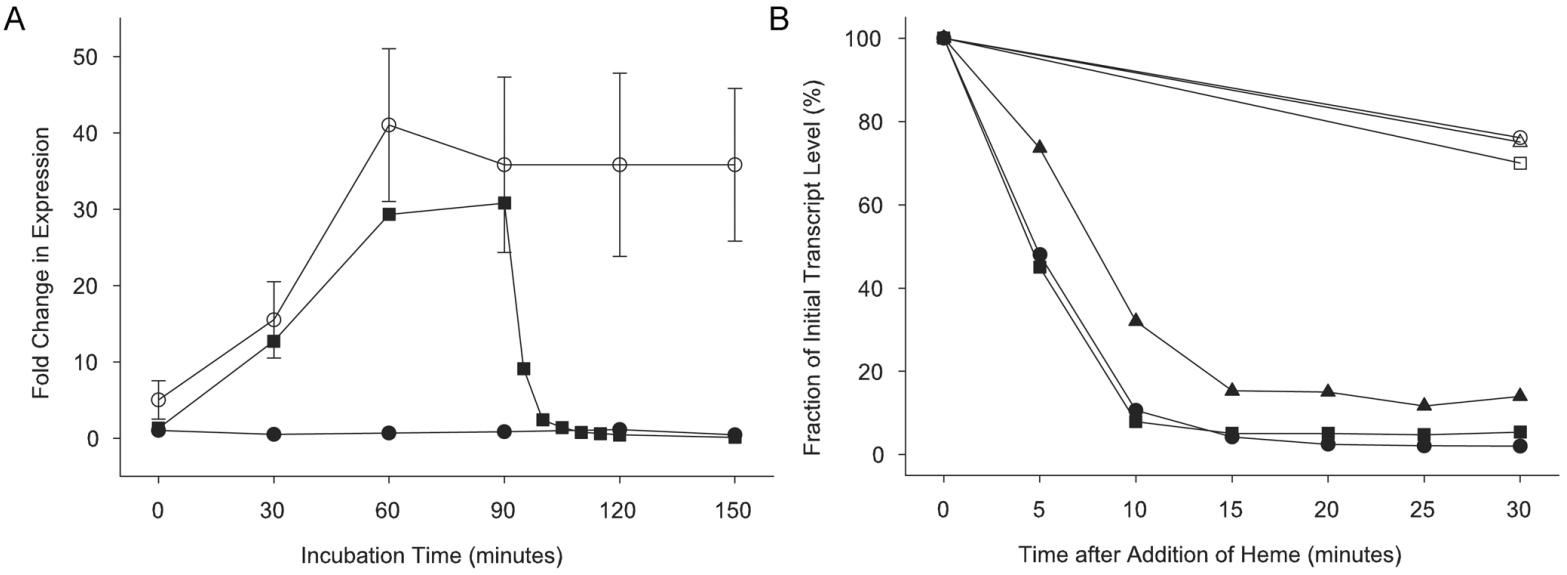
**Repression kinetics defining the window of transcriptional regulation for specified FeHm-regulated genes in strain Hib 10810**. (A) Fold changes in expression of *hitA *in isolate Hib 10810 over the course of 150 minutes of growth under three different sets of conditions. Strain Hib 10810 was grown in either: 1) medium that was restricted for iron and heme for the duration of the experiment (open circles), 2) medium that was fully supplemented with iron and heme for the duration of the experiment (closed circles) or 3) medium that was restricted for iron and heme up to 90 minutes at which point iron and heme were added to fully supplement the medium (closed squares). (B) Fraction of transcript level remaining relative to the transcript level seen at the 90 minute timepoint in panel A. Results are shown for transcript levels of: *hitA *in medium fully supplemented with iron and heme for the duration of the experiment (open squares); *hitA *in medium that was restricted for iron and heme up to 90 minutes at which point iron and heme were added to fully supplement the medium (closed squares); *tbp1 *in medium fully supplemented with iron and heme for the duration of the experiment (open circles); *tbp1 *in medium that was restricted for iron and heme up to 90 minutes at which point iron and heme were added to fully supplement the medium (closed circles); *hxuC *in medium fully supplemented with iron and heme for the duration of the experiment (open triangles); *hxuC *in medium that was restricted for iron and heme up to 90 minutes at which point iron and heme were added to fully supplement the medium (closed triangles).

Having determined the time course and conditions of maximal transcription of the three FeHm-repressible genes in the two clinical isolates, we next examined the time course of repression of transcription of these same genes in order to determine the minimal time window required to detect maximal differential regulation. The kinetics of repression of *hitA*, *tbp1 *and *hxuC *by FeHm were determined in the following way for each clinical isolate. Three flasks were prepared and inoculated with a given strain. Two of these flasks contained FeHm-restricted media (i.e. BHI with no added heme and additionally supplemented with deferroxamine to chelate iron) while the third flask was FeHm supplemented (i.e. BHI with 0.5 mM FeCl_3 _and 10 μg/ml heme). Each set of flasks was inoculated with the respective isolate, and samples (500 μl) taken at 30 minute intervals for RNA isolation and Q-PCR analysis. After 90 minutes of incubation, FeHm (0.5 mM FeCl_3_,10 μg/ml heme) was added to one of the flasks containing FeHm-restricted media and samples were removed at 5 minute intervals from each of the three flasks for RNA isolation. Between flask comparisons of the *hitA, tbp1 *and *hxuC *transcript levels determined by Q-PCR analysis demonstrated that marked differences were associated with the timing of FeHm supplementation. FeHm supplementation from the outset led to no increase in transcripts over the duration of the experiment. Conversely, the FeHm-restricted cultures displayed increased transcription of *hitA, tbp1 *and *hxuC *consistent with that observed in the previous experiment. In the FeHm-restricted environment the transcription *of hitA*, *tbp1 *and *hxuC *increased with a similar profile under the two conditions, until the addition of exogenous FeHm at 90 minutes at which point a very rapid decrease in the transcript levels was observed. Within 20 minutes following this addition the transcript levels of genes under this condition were indistinguishable from those in cultures containing FeHm throughout. For simplicity, Figure 3A shows only the data for the fold changes in transcripts of the *hitA *gene from Hib 10810 over the course of a representative experiment. Transcripts of *tbp1 *and *hxuC *displayed similar profiles (data not shown). Figure 3B shows the rapid reduction in transcripts for the three genes, post FeHm supplementation, for strain 10810 cultures. Transcripts for these three genes in the isolate NTHi R2866 showed a similar profile (data not shown).

A necessary control experiment provided assurance that the various conditions of FeHm supplementation employed in the previous experiment altered transcription levels in a gene- and effector-specific manner. The *ompP2 *gene was chosen for this analysis because it was known to be constitutively transcribed and thus not expected to be regulated by FeHm. Consistent with these expectations, *ompP2 *transcript levels did not differ in cells grown under the same FeHm conditions described in Figure 3 (data not shown). Viable counts showed that cultures grown under these conditions also did not differ (data not shown).

Taken together, the results described above establish that the profiles of growth, transcriptional regulation by FeHm, kinetics of repression and derepression of marker genes known to be FeHm repressible, and specificity of the FeHm effect on transcript levels in the two clinical isolates are highly similar to those previously reported for strain Rd KW20 [25]. These findings permit the use of the same experimental conditions for microarray analysis of the FeHm modulon of strains Hib 10810 and NTHi R2866 that we had successfully used in our genome wide transcriptional analysis of strain Rd KW20.

### Microarray analysis of the FeHm modulons of *H. influenzae *10810 and R2866

The culture and FeHm supplementation regimen used to obtain paired specimens for microarray analysis was based on the preceding findings. Triplicate cultures of each clinical isolate were prepared and sampled in the following way. Three flasks containing 120 ml FeHm-restricted media were inoculated per isolate. Following a 90 minute incubation under derepressing conditions, a 60 ml sample was removed from each for RNA purification. Immediately following removal of this sample, each flask was supplemented with FeHm (0.5 mM FeCl_3_,10 μg/ml heme) to establish repressing conditions. After a further 20 minute incubation period under repressing conditions, a second 60 ml sample was removed for RNA purification.

This experimental design based upon the empirically established kinetics of repression of three FeHm-repressible genes, allowed us to use a short validated repression time window of 20 minutes to compare the fully derepressed and repressed states. This short time window over which samples comparing the two conditions were obtained was expected to minimize non-specific/secondary effects of differences in FeHm availability.

**Figure 4:**
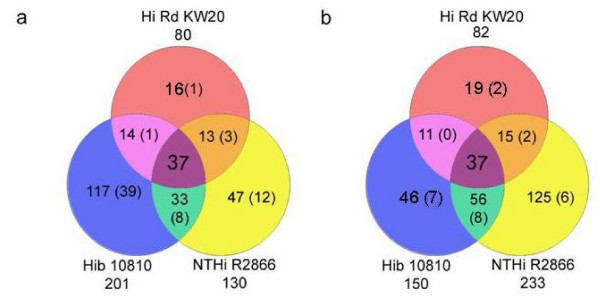
**Comparison of the FeHm-regulated genes in Rd KW20, NTHi R2866 and Hib 10810**. (A) Genes that are repressible by iron and heme. (B) Genes that are inducible by iron and heme. The number under each isolate name indicates the total number of genes determined to be regulated for that isolate under the appropriate conditions. Numbers in parentheses indicate the number of genes that are regulated that are distinct to those isolates i.e. the gene is not present in the genome of the other isolate(s). The data show that there is a core set of 37 genes that are present in and regulated in all three isolates for both the FeHm-repressible and the FeHm-inducible gene sets.

Approximately 50–60 μg total RNA was purified from each experimental sample. The RNA samples were analyzed for quality by gel electrophoresis; for all samples, distinct bands were observed (data not shown). The absence of contaminating DNA was confirmed by PCR using oligonucleotide primers targeting the 16S rRNA and *hitA *genes (data not shown). Samples were submitted to NimbleGen Inc., where they underwent in-house quality control prior to being processed for microarray hybridizations. Following hybridization, analysis of the resulting data showed that of the 1820 ORFs on the array corresponding to R2866 genes, 363 (20%) were differentially transcribed in a statistically significant manner (Additional File 1: Comparison of genes identified as FeHm regulated in isolates NTHi R2866, Hib 10810 and Rd KW20 and Additional File 2: Fold transcriptional change of NTHi R2866 genes following supplementation of FeHm restricted media with exogenous FeHm). Of these 363 genes, 130 (7%) were preferentially transcribed in FeHm-restricted conditions and 233 (13%) were maximally transcribed in FeHm-replete conditions. Of the 1883 ORFS represented on the array that correspond to Hib 10810 genes, 353 (19%) were significantly differentially transcribed. Of these 351 genes, 203 (11%) were maximally transcribed in FeHm-restricted conditions and 150 (8%) in FeHm-replete conditions (Additional File 1: Comparison of genes identified as FeHm regulated in isolates NTHi R2866, Hib 10810 and Rd KW20, and Additional File 3: Fold transcriptional change of Hib 10810 genes following supplementation of FeHm restricted media with exogenous FeHm). In both sets of microarray data, transcripts of the FeHm repressible genes *tbp1*, *hitA *and *hxuC *were elevated under FeHm restriction while the constitutive *ompP2 *gene transcripts showed no significant changes for either isolate, as expected from the preliminary Q-PCR experiments. (For simplicity, genes preferentially transcribed in FeHm-restricted media or FeHm-replete media will now be referred to as FeHm negative (FeHm-ve) or FeHm positive (FeHm+ve) respectively). Comparison of the microarray data from Hib 10810 and NTHi R2866 with that derived from Rd KW20 shows that 37 genes are differentially expressed in FeHm-replete conditions and a separate 37 genes are differentially expressed in FeHm-deplete conditions in all 3 isolates (Figure 4).

### Validation of the microarray data

Several genes were selected for analysis by Q-PCR for validation of the microarray data. These Q-PCR analyses were performed on the same RNA samples used for the microarray analysis. Primers used for these analyses are shown in Additional File 4: Primers used for Q-PCR analysis. Genes analyzed included examples of those that were: 1) preferentially expressed under FeHm limitation in both isolates, 2) preferentially expressed under FeHm supplementation in both isolates and 3) those genes that did not exhibit significant fold changes in either isolate. Genes analyzed that were preferentially expressed under FeHm limitation (FeHm-ve) were: HI0017 which encodes formate acetyltransferase, HI0591 encoding ornithine decarboxylase, HI0997m encoding a putative outer membrane protein, HI1349 which encodes a probable DPS ferritin, HI1427 encoding a putative ABC transporter, the transferrin binding protein gene *tbp1 *(HI0994) and the heme-hemopexin utilization gene *hxuC *(HI0262). Genes selected for validation that were preferentially expressed under FeHm supplementation (FeHm+ve) were: HI0007 which encodes a putative formate dehydrogenase, HI0185 encoding alcohol dehydrogenase, HI0980 encoding the DNA architectural protein Fis, HI1384 encoding the ferritin subunit A1 and HI1706 which encodes the osmoprotection-related protein BetT. Genes selected for validation which failed to show significant fold change when analyzed by microarray were: HI0502 which encodes RbsG (involved in ribose transport) and HI1368 *pqqL*, encoding a putative protease.

Comparison of this Q-PCR data with the array data (Table 1) shows 100% agreement between regulatory status based on the fold differences observed from the array and from the Q-PCR in both isolates, thus validating the array data.

**Table 1:** Array validation by Q-PCR

	**NTHi R2866**		**Hib 10810**	
**Gene**	**Array^a^**	**Q-PCR^b^**	**Array^a^**	**Q-PCR^b^**

HI0007 *fdxI*	+7.49	+9.88	+6.00	+5.20
HI0017 *yfiD*	-8.25	-10.72	-3.03	-3.34
HI0185 *adhC*	+5.86	+7.65	+4.01	+6.52
HI0262 *hxuC*	-24.69	-32.65	-4.03	-12.12
HI0502 *rbsA*	ns	+1.08	ns^c^	+1.13
HI0591 *potE*	-11.40	-13.39	-13.78	-2.91
HI0980 *fis*	+4.67	+5.99	+2.56	+5.19
HI0994 *tbp1*	-9.56	-10.87	-8.16	-16.53
HI0997m *ompU*	-15.68	-4.43	-14.61	-4.21
HI1349 *dps*	-4.12	-3.03	-3.70	-4.81
HI1368 *pqqL*	ns	+1.67	ns	-1.60
HI1384 *ftnA1*	+3.21	+4.34	+2.01	+4.43
HI1427 ABCt.	-6.60	-5.18	-2.32	-2.64
HI1706 *betT*	+3.24	+3.23	+3.39	+3.57

^a^Fold change between the FeHm unsupplemented and supplemented sample from the microarray.

^b^Fold change between the FeHm unsupplemented and supplemented sample from the Q-PCR analysis.

^c^ns, not significant

### Confirmation of the core FeHm modulon

Examination of the microarray data for Hib 10810 and NTHi R2866 and the comparison with the published Rd KW20 data (Additional File 1: Comparison of genes identified as FeHm regulated in isolates NTHi R2866, Hib 10810 and Rd KW20) show that each gene falls into one of three distinct sets: 1) those differentially regulated in a statistically significant manner in all isolates; 2) those variably regulated across isolates; 3) those not regulated (and thus excluded from Additional File 1). The former set of genes comprise the putative core FeHm modulon while the second may be isolate specific responses to FeHm status or results of experimental variation. To confirm that the profiles we have observed are reproducible we performed an independent experiment that repeated the transcription-repression protocol detailed above, but performed Q-PCR analysis on the samples (as opposed to microarray) using select primer sets targeting representative genes (Additional file 4: Primers used for Q-PCR analysis). Since our focus is elucidation of genes preferentially transcribed under FeHm restriction, we primarily selected targets from the FeHm-ve genes. Each isolate was grown as described for the microarray experiments (for details pertaining to growth of Rd KW20 see Whitby *et al *2006 [25]) and samples taken at 90 and 110 minutes (20 minutes post FeHm supplementation of the FeHm restricted media). Following Q-PCR, genes with a fold change ≥ 1.5 were considered as differentially regulated between the two growth conditions. Fifty two targets were selected from the genes comprising the putative core modulon (genes displaying a fold change ≥ 1.5 by microarray in all 3 isolates). These were selected to target the monocistronic genes and at least one gene in the majority of operons. Each target was examined in each isolate. Of the 156 Q-PCR results, approximately 94% (9 mismatches) matched that of the microarray in terms of regulatory status. The actual data values for each PCR, compared to that of the microarray experiment are shown in Table 2. These data show that the regulation of the core FeHm modulon is reproducible in each of the three isolates. In addition, these data provide strong evidence for conservation of the core FeHm modulon across the species and in other FeHm-restricted environments such as the human host.

**Table 2:** Reproducibility of expression of core FeHm stimulon genes

	**NTHi R2866**		**Hib 10810**		**Rd KW20**	
	
**Locus^a^**	**Array^b^**	**QPCR^c^**	**Array^b^**	**QPCR^c^**	**Array^d^**	**QPCR^c^**
HI0006m	**8.36**	**12.99**	**7.58**	**18.00**	**not on**^†^	**9.76**
HI0007	**7.49**	**11.02**	**6.00**	**9.31**	**3.40**	**7.29**
HI0017	**-8.25**	**-19.19**	**-3.03**	**-14.92**	**-1.87**	**-8.08**
HI0020	**-2.54**	**-4.81**	**-1.83**	**-6.04**	**-1.75**	**-2.29**
HI0035	-2.46	-1.07	**-1.74**	**-1.71**	**-1.55**	**-2.55**
HI0075	-2.95	1.03	**-2.54**	**-3.26**	**-4.03**	**-10.03**
HI0092	**-7.26**	**-2.14**	**-2.23**	**-1.51**	**-2.04**	**-5.51**
HI0095	**-12.56**	**-4.63**	**-3.93**	**-7.42**	**-10.21**	**-23.17**
HI0097	**-14.27**	**-38.00**	**-4.52**	**-67.60**	**-8.15**	**-39.73**
HI0113	**-4.72**	**-2.06**	**-2.28**	**-4.77**	**-2.36**	**-2.56**
HI0157	**2.18**	**3.29**	**1.52**	**3.71**	**1.55**	**3.64**
HI0173	**3.07**	**3.54**	**1.68**	**2.23**	**1.90**	**2.32**
HI0185	**5.86**	**38.95**	**4.01**	**41.06**	**8.04**	**30.77**
HI0206	**-2.25**	**-2.18**	**-2.09**	**-4.89**	**-1.60**	**-2.53**
HI0230	**2.91**	**5.42**	**2.51**	**3.10**	**2.31**	**2.67**
HI0244	**2.59**	**10.47**	**1.87**	**3.13**	**2.30**	**4.51**
HI0251	*-1.99*	*1.18*	**-1.69**	**-3.12**	*-2.75*	*-1.16*
HI0252	*-2.45*	*1.15*	**-1.87**	**-3.90**	**-3.02**	**-2.56**
HI0257	**-2.27**	**-5.52**	**-1.52**	**-8.86**	**-1.61**	**-2.32**
HI0262	**-24.69**	**-13.38**	**-4.03**	**-5.95**	**-10.30**	**-26.93**
HI0263	**-29.31**	**-38.82**	**-3.29**	**-8.69**	**-9.20**	**-25.57**
HI0319	**2.76**	**4.47**	**1.54**	**2.83**	**1.96**	**2.88**
HI0324	**2.04**	**4.58**	**1.80**	**2.00**	**1.63**	**2.34**
HI0584	**-1.84**	**-1.83**	**-1.59**	**-2.09**	**-1.69**	**-3.39**
HI0591	**-11.40**	**-11.89**	**-13.78**	**-7.63**	**-3.84**	**-4.78**
HI0623	**2.51**	**8.29**	**2.07**	**3.76**	**1.62**	**2.75**
HI0669	*-2.08*	*1.02*	**-1.72**	**-1.98**	**-1.67**	**-2.18**
HI0689	**-4.96**	**-3.71**	**-1.85**	**-2.94**	**-1.53**	**-2.93**
HI0691	**-5.71**	**-3.95**	**-3.40**	**-7.87**	**-1.61**	**-1.98**
HI0809	**-6.41**	**-2.41**	**-3.16**	**-3.58**	**-2.87**	**-5.29**
HI0863	**-2.00**	**-1.66**	**-1.88**	**-1.57**	*-1.51*	*-1.39*
HI0864	**1.89**	**7.42**	**1.91**	**4.39**	**1.71**	**3.97**
HI0878	**1.70**	**4.99**	**2.00**	**3.80**	**1.96**	**2.55**
HI0980	**4.67**	**6.68**	**2.56**	**5.56**	**2.06**	**7.82**
HI0994	**-9.56**	**-3.83**	**-8.16**	**-26.02**	**-12.40**	**-23.55**
HI0997m	**-15.68**	**-18.62**	**-14.61**	**-11.49**	**not on**^†^	**-10.70**
HI0999	**1.66**	**5.65**	**1.62**	**2.75**	**1.68**	**3.99**
HI1051	**3.02**	**6.10**	**2.44**	**3.16**	**1.80**	**4.32**
HI1069	**8.33**	**5.87**	**2.53**	**6.96**	**2.13**	**3.89**
HI1094	*2.53*	*1.44*	**1.60**	**1.94**	**1.81**	**1.66**
HI1174m	**-2.06**	**-1.93**	**-1.75**	**-3.22**	**not on**^†^	**-2.78**
HI1210	**-5.29**	**-3.18**	**-2.68**	**-6.57**	**-2.47**	**-4.41**
HI1214	**1.92**	**2.21**	**2.40**	**2.33**	**1.74**	**2.99**
HI1228	**-3.35**	**-2.91**	**-1.50**	**-2.55**	**-1.62**	**-1.73**
HI1282	**2.87**	**6.73**	**3.13**	**4.16**	**1.96**	**6.62**
HI1349	**-4.12**	**-7.53**	**-3.70**	**-25.61**	**-1.53**	**-5.48**
HI1359	**-3.28**	**-2.74**	**-3.10**	**-5.00**	**-1.73**	**-2.49**
HI1384	**3.21**	**9.31**	**2.01**	**5.38**	**2.60**	**4.76**
HI1398	**-5.25**	**-3.55**	**-2.98**	**-5.80**	**-2.32**	**-3.82**
HI1427	*-6.60*	*-1.42*	**-2.32**	**-1.90**	**-6.12**	**-11.91**
HI1706	**3.24**	**14.28**	**3.39**	**5.81**	**3.14**	**12.76**
HI1733	**3.23**	**7.99**	**2.89**	**3.27**	**1.81**	**2.71**

^a ^The gene locus in Rd KW20

^b ^Fold change between the FeHm unsupplemented and supplemented sample from the microarray

^c ^Fold change between the FeHm unsupplemented and supplemented sample by Q-PCR of an independent experiment.

^d ^Fold change between the FeHm unsupplemented and supplemented sample by Q-PCR of the Rd KW20 previously published by Whitby et al. [25]

^† ^not present on Rd array, but inclusion in the core verified by Q-PCR of array samples

Values in plain italic text show a disagreement in regulatory status between the microarray and Q-PCR data.

With regard to genes showing potential different regulatory profiles between isolates, 53 genes were selected comprising 39 that were FeHm-ve in at least one of the isolates and 14 that were FeHm+ve in at least one isolate. Of these, a significant portion had data values determined to be below our threshold of significance by microarray. Q-PCR was performed on all isolates to either confirm the known microarray value or determine the value (for those deemed non-significant). For genes with a significant value 69% (67 of 98) of the Q-PCRs resulted in agreement of regulatory status (Table 3). Of the non-significant values (by microarray) 62% (36 of 58) were returned by Q-PCR to be below 1.5 fold difference. Thirteen of the 22 Q-PCRs that showed a >1.5 fold difference (for the previously determined "non-significant" genes) resulted in that gene displaying a regulated profile in all three isolates. Interestingly, several of these genes are internal to an operon for which the other genes were already deemed part of the FeHm core modulon. For example, the genes HI0098-HI0099 (*hitB *and *hitC*) were shown by microarray to be regulated in 10810 and Rd KW20 but not R2866, although the first gene in the operon HI0097 (*hitA*) was regulated in all three strains. Q-PCR analysis confirmed regulation of these genes in all 3 isolates.

**Table 3:** Reproducibility of expression of non-core FeHm stimulon genes

	**NTHi R2866**		**Hib 10810**		**Rd KW20**	
	
**Locus^a^**	**Array^b^**	**QPCR^c^**	**Array^b^**	**QPCR^c^**	**Array^d^**	**QPCR^c^**
HI0098	**ns**	**-1.56**	**-2.70**	**-2.80**	**-3.55**	**-4.28**
HI0099	**ns**	**-2.69**	**-2.16**	**-2.48**	**-3.56**	**-3.82**
HI0152	**ns**	**1.47**	*-2.12*	*-1.44*	**-1.08**	**-1.13**
HI0153m	*-2.70*	*1.10*	**-2.24**	**-2.02**	**not on**	**-1.38**
HI0164	**ns**	**4.02**	*1.26*	*2.01*	**1.63**	**1.98**
HI0223	**ns**	**-1.33**	**ns**	**1.10**	**-2.45**	**-4.70**
HI0246	**ns**	**-1.75**	**-1.94**	**-4.29**	**ns**	**-1.20**
HI0254	**ns**	**-1.10**	**-2.56**	**-2.41**	*-1.43*	*-2.22*
HI0298	**ns**	**1.14**	*-1.97*	*-1.08*	**ns**	**-1.25**
HI0342	**3.21**	**4.7**	**ns**	**2.20**	**not on**^†^	**1.54**
HI0361	**-7.89**	**-3.14**	**ns**	**-1.30**	**-8.91**	**-15.27**
HI0362	**-6.01**	**-7.29**	*-1.17*	*-2.96*	**-11.75**	**-31.98**
HI0365	**2.18**	**6.68**	**1.91**	**4.19**	**1.38**	**3.77**
HI0502	**ns**	**-1.19**	**ns**	**-1.67**	**-1.60**	**-11.24**
HI0507	**ns**	**16.41**	**2.27**	**10.47**	**2.04**	**5.76**
HI0534	**ns**	**1.31**	**-3.38**	**-9.02**	**-3.12**	**-5.49**
HI0563	**ns**	**-1.1**	*-1.51*	*-1.03*	**ns**	**-1.23**
HI0601	*-1.87*	*1.69*	**ns**	**-1.16**	*-1.66*	*1.22*
HI0661	**-6.52**	**-2.36**	**-4.14**	**-7.08**	*-1.20*	*-1.59*
HI0663m	**ns**	**1.08**	**-2.11**	**-1.98**	**-1.61**	**-1.87**
HI0670	**ns**	**1.27**	**-1.74**	**-1.50**	**-1.60**	**-1.74**
HI0682	**-5.03**	**-7.47**	**-4.18**	**-16.92**	*1.54*	*-8.05*
HI0752	**ns**	**1.23**	*-1.56*	*-1.49*	**ns**	**1.01**
HI0774	**ns**	**1.22**	**-2.31**	**-1.30**	**ns**	**-1.26**
HI0811	*-2.20*	*-1.07*	**ns**	**1.06**	**ns**	**1.45**
HI0833	**ns**	**-1.25**	*-1.64*	*-1.25*	**ns**	**1.64**
HI0845	**ns**	**1.20**	**-1.51**	**-1.65**	**ns**	**1.03**
HI0889	**1.83**	**5.82**	**1.68**	**4.90**	*-1.09*	*2.99*
HI0890m	**2.50**	**4.60**	**3.48**	**7.49**	**ns**	**3.20**
HI1010	**ns**	**5.52**	**1.91**	**12.00**	*1.86*	*1.32*
HI1047	**ns**	**-1.4**	**-4.99**	**-2.58**	*-1.10*	*1.86*
HI1064m	**ns**	**1.09**	*-1.57*	*-1.31*	**not on**	**-1.45**
HI1107	**ns**	**-5.42**	**-2.76**	**-3.42**	*-1.08*	*-1.66*
HI1173	**ns**	**2.24**	**2.93**	**1.62**	**1.52**	**1.88**
HI1180	*-5.68*	*-1.44*	*-2.47*	*-1.24*	**-1.20**	**1.42**
HI1190	**ns**	**-1.01**	**-1.60**	**-1.72**	**-1.92**	**-1.73**
HI1217	*-2.75*	*1.1*	*-1.48*	*-2.20*	**-2.99**	**-3.60**
HI1218	**4.67**	**33.45**	**3.58**	**17.21**	**ns**	**11.58**
HI1224	*-1.66*	*-1.2*	**-1.68**	**-1.93**	**ns**	**1.13**
HI1275	*-2.01*	*1.07*	**-1.37**	**1.01**	**-3.34**	**-5.77**
HI1305	**ns**	**2.29**	**1.68**	**2.29**	**ns**	**1.71**
HI1356	**ns**	**-1.83**	**-2.25**	**-1.83**	**-1.82**	**-2.15**
HI1366	*-1.66*	*-1.08*	**-1.59**	**-1.60**	**ns**	**-1.42**
HI1368	**ns**	**2.24**	**ns**	**1.29**	**-1.86**	**-2.42**
HI1383m	**ns**	**-1.15**	**-1.78**	**-2.63**	**ns**	**-1.72**
HI1444	**5.19**	**13.43**	**3.47**	**11.77**	**ns**	**7.21**
HI1455	**ns**	**-2.24**	*-2.42*	*-1.23*	**ns**	**1.16**
HI1546	**ns**	**1.57**	*-1.74*	*1.10*	**ns**	**1.38**
HI1607	**2.30**	**2.08**	**ns**	**1.28**	*1.09*	*2.17*
HI1612	**2.27**	**2.41**	*1.50*	*-1.24*	*1.23*	*4.77*
HI1693	**ns**	**-1.1**	*-1.52*	*-1.47*	**ns**	**-1.11**
HI1738	**2.09**	**5.82**	**2.52**	**3.28**	**ns**	**2.38**
*tnaA*^e^	**-16.22**	**-3.81**	**-4.93**	**-5.85**	**n/a**	**n/a**

^a ^The gene locus in Rd KW20

^b ^Fold change between the FeHm unsupplemented and supplemented sample from the microarray

^c ^Fold change between the FeHm unsupplemented and supplemented sample by Q-PCR of an independent experiment.

^d ^Fold change between the FeHm unsupplemented and supplemented sample by Q-PCR of the Rd KW20 previously published by Whitby et al. [25]

^e ^gene R2866v6.1850c- and Hib 10810.0830 in the R2866 and Hib 10810 genome respectively.

^† ^not present on Rd array, but regulation verified by Q-PCR of the original Rd KW20 array samples.

n/a – gene not present in this isolate

Values in plain italic text show a disagreement in regulatory status between the microarray and Q-PCR data.

### Operonic concordance

Additional File 1 shows the correlation of responsive loci in the two strains analyzed in this study and in the previously reported analysis of strain Rd KW20. From this table it is clear that genes exhibiting different regulation between the isolates are coregulated with the rest of that operon within the isolate (where that gene is part of an operon). The fact that there is operonic concordance in each sample set attests to the utility of the novel multi-genome chip we designed for these studies.

### The core FeHm modulon contains 37 genes preferentially expressed under FeHm limitation

As expected, the core FeHm-ve genes identified by microarray include many genes known to be associated with FeHm acquisition. These included *hitA, tonB, exbD, exbB, hgpB, hgpC *and the *hxu *and *tbp operons*. In addition to genes clearly or potentially associated with FeHm acquisition we identified several other loci with roles potentially related to FeHm metabolism such as protection against oxidative stress, FeHm storage and detoxification and biofilm formation.

One such gene, HI1349, has similarities to DPS ferritins (DNA Protecting protein under Starved conditions) which non specifically bind DNA to protect from damage by reactive oxygen species [30]. The DPS ferritins in other bacteria are induced by nutritional starvation, including metal ion starvation [31]. Recently the expression of the *H. influenzae *DPS ferritin has been shown to be under the control of both the ArcAB two-component regulatory system [32] and OxyR [33]. The ArcAB regulatory system controls metabolic flux during anaerobic growth and also appears to control genes in a pre-emptive protection against a burst of reactive oxygen intermediates (ROI) when aerobic growth resumes [32]. Mutation of *arcA *decreases the expression of the DPS ferritin. Furthermore, the DPS ferritin was shown to mediate resistance to peroxide [32]. The OxyR regulon is the coordinated response to oxidative damage induced by treatment with peroxide [33]. The increased expression of the DPS ferritins under FeHm limitation may similarly protect against ROI should exogenous FeHm be added to the culture media.

The HI0590-591 operon, encoding putrescine-ornithine antiporter and ornithine decarboxylase (*potE *and *speF *respectively), is also similarly regulated in all isolates. This locus is potentially important in virulence since polyamines play a major role in the regulation of numerous cellular processes by modulating the biosynthesis of DNA, RNA and proteins [34-36]. In *Yersinia pestis*, polyamines play an essential role in biofilm formation [37], and in *E. coli *they have been implicated in defense against peroxide [38]. Polyamines have also been implicated as an alternative source of energy production via generation of a transmembrane proton motive force [39]. Recently, this locus has been shown to be regulated by ArcA in *H. influenzae *[32].

Other FeHm-ve genes include those encoding phosphoenolpyruvate carboxykinase (*pckA*, HI0809) fumerase C (*fumC *HI1398) and malate dehydrogenase (HI1210). Together with the product of HI0534 encoding aspartate ammonia lyase (*aspA *-regulated only in Hib10810) and asparaginase B (*ansB *HI0745- regulated only in Hib10810), these enzymes would lead to the sequential conversion of L-asparagine to L-aspartate, fumarate, malate, oxaloacetate and finally, with the action of PckA, yield phosphoenolpyruvate which then feeds into gluconeogenesis to generate a pool of glucose phosphate. It is interesting that the NTHi R2866 genome lacks HI0745 (*ansB*) and thus would not create the substrate of *aspA *(HI0534), a gene which it may not up-regulate.

Our previous study to determine the FeHm modulon of Rd KW20 elucidated several FeHm-ve genes with a potential involvement in FeHm acquisition [25]. Included in these genes were two that encoded putative TonB-dependent outer membrane proteins (genes HI0113 and HI1369). This study confirms the increased transcription of these genes in two further isolates while under FeHm limitation. The role of these loci is currently unknown; however, the sequence homologies and similarities in the predicted secondary structure to other known TonB-dependent proteins suggest a role in iron and/or heme acquisition. Both HI0113 and HI1369 are foci of ongoing research with respect to their potential roles in FeHm acquisition.

### The core FeHm modulon contains 37 genes preferentially expressed under FeHm supplementation

Included in the subset of FeHm+ve genes are several genes with a clear role in FeHm homeostasis and resistance to reactive radicals as well as genes for which the encoded protein requires iron or heme for functionality. The genes HI1384 and HI1385 encode the ferritin subunits which form a macromolecular structure that stores and detoxifies Fe when cellular levels become elevated. Also in the FeHm+ve group are genes HI0006m-HI0009 comprising the formate dehydrogenase complex which is an Fe-S cluster containing system, and the genes in the operon HI0172-HI0174 which encode an Fe-S assembly complex. Interestingly, the formate dehydrogenase locus (HI0006m-HI0009) is also part of the ArcAB regulon and shows decreased transcription in the *arcA *mutant [32]. The genes HI1066-HI1069 encode subunits of nitrite reductase, which may assist in defense against reactive nitrogen species.

### Not all genes involved in FeHm acquisition are contained in the core FeHm modulon

A locus identified in the current study, yet missed in the Rd KW20 microarray, is that of gene HI0997m. In NTHi R2866 and Hib 10810 it is the 5^th ^and 2^nd ^most upregulated gene respectively. Regulation of this gene was not observed in the Rd KW20 array study since it was excluded from the chip design. This exclusion was due to the presence of a stop codon within the reading frame of this gene in the original published Rd KW20 genomic sequence (codon 147 of 481) [40]. The exclusion of this and other such sequences on the Rd KW20 chip is one of the driving forces behind the development of new microarrays targeting all genes and pseudogenes of the isolates being studied. The product of HI0997m exhibits homology to a putative outer membrane protein, OmpU, of *Neisseria meningitides*. This protein has been implicated in heme utilization since it facilitates the uptake of exogenous heme in *Esherichia coli *(see comments in the Entrez nucleotide entry for *N. meningitidis ompU*, Accession number AF118122). We used Q-PCR, to examine the FeHm regulated transcription of the region immediately upstream of the internal stop codon in HI0997m of strain Rd KW20 using cDNA derived from the samples detailed in our previous Rd KW20 microarray study [25]. Transcription of this region of HI0997m in Rd KW20 under FeHm limitation was 6.15 fold higher than observed in FeHm-replete media. Thus, HI0997m exhibits a similar pattern of transcriptional change in response to FeHm limitation in all three strains and may thus warrant inclusion as a core FeHm-ve gene. However, nucleotide sequencing of three independent PCRs of HI0997m from strain Rd KW20 confirmed the presence of the internal stop codon, and thus HI0997m in Rd KW20 may not encode a functional protein (data not shown).

An additional locus that was identified in our previous study, and confirmed in this report, is that of the 4 gene operon HI0359-HI0362. This locus has homology to the *yfeABCD *locus of *Yersinia pestis *which plays a role in the uptake Fe, Mn and Zn [41]. From the microarray data, all genes in this locus are clearly upregulated in both Rd KW20 and R2866 yet appear to be unregulated in Hib strain 10810. To confirm this, Q-PCR was performed in the first and second genes HI0362 and HI0361 respectively (Table 3). The results show that the first gene of the operon is upregulated in each isolate, while the second gene in Hib 10810 is not, confirming the differential regulation observed by microarray.

**Figure 5:**
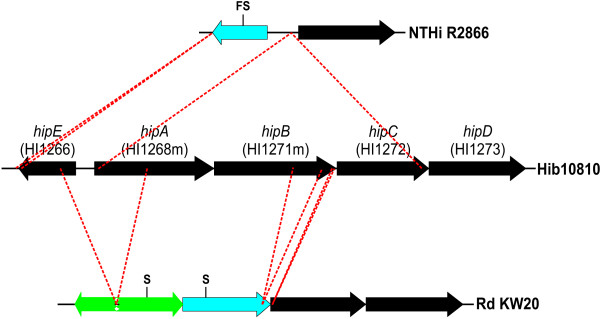
**The *hip *locus in *H. influenzae *strains R2866, 10810 and Rd KW20**. The locus number designations are those from the Rd KW20 sequence. Genes colored blue represent substantially intact genes containing a frameshift or nonsense mutation. Genes colored green represent substantially truncated genes lacking promoter regions. The *hip *locus encodes a hypothetical protein (*hipE*), a probable ABC transport system (*hipABC*) with homology to heme transporters in other organism, and a probable methyltransferase (*hipD*). The intact locus is found in 10810 and other type b capsulated strains while various deletions are present in NTHi strains (data not shown). In R2866, two apparent deletions have occurred; a 38 bp deletion at the distal end of *hipE *and a 2676 bp deletion inclusive of *hipABC*. The *hipE *gene is also frameshifted (site marked by "FS"). In Rd KW20, three apparent deletions have occurred; a 720 bp deletion has removed the proximal regions of *hipE *and *hipA*, including the likely promoter control elements, and 237 bp and 19 bp deletions have removed portions of *hipB*. In addition, stop codons are present in the remaining *hipA *and *hipB *sequences (designated with an "S")

The three gene operon comprising HI0126-HI0131 is FeHm-ve in Rd KW20 and R2866 but absent from the genome of Hib 10810. These three genes have homology to the iron repressible *afu *locus of *A. pleuropneumoniae *[42,43] as well as the SfuABC proteins of *Serratia marcescens *[44] and the YfuABC proteins of *Y. pestis *[45]. This system constitutes a periplasmic binding protein-dependent iron transport system in these organisms. The three genes encode the periplasmic iron-binding protein (AfuA), a highly hydrophobic integral cytoplasmic membrane protein with two consensus permease motifs (AfuB) and a hydrophilic peripheral cytoplasmic membrane protein with ATP-binding motifs (AfuC), respectively. Also included in genes lacking an intact homologue in the other two isolates is the strain 10810 locus designated Hib10810.1401. This FeHm-repressible gene encodes a heme internalization protein designated HipA [46]. The other strains examined have various genomic deletions at the site of this gene. In Rd KW20 the homologous CDS to *hipA *is HI1268m. Comparison of Rd KW20 with the Hib 10810 *hip *locus indicates that Rd KW20 lacks the region spanning the middle of the upstream, divergently transcribed, HI1266 (*hipE*) gene to the middle of the *hipA *sequence, including the intergenic region, effectively leaving both fragments of HI1266 and *hipA *without promotors. The downstream genes in Rd KW20, *hipBCD*, are partially intact, but show no regulation by FeHm levels, possibly due to the lack of any promoter elements. Conversely, the isolate NTHi R2866 has a deletion spanning *hipABC*, leaving the gene HI1266 intact. Even though the upstream region of the *hipA *gene remains, there is no apparent regulation of the HI1273 gene. Comparison of the deletions at this locus in the three strains is depicted in Figure 5.

The genes identified here with potential roles in heme and iron acquisition are each the subject of ongoing studies in this laboratory as we seek to further define the range and role of FeHm acquisition systems in *H. influenzae*.

### FeHm levels may modulate transcription of several genes previously defined as members of the ArcA regulon

The data discussed above details a number of genes that are also regulated as part of the ArcA regulon [32] (the ArcA studies were performed in an *H. influenzae *strain Rd derivative, although it is unclear if the isolate is identical to the ATCC strain Rd KW20 used in our previous microarray study). The presence of several FeHm regulated genes discussed above in the ArcA regulon led us to compare the other genes of the ArcA regulon and those of the FeHm modulon reported here. Table 4 shows the genes reported to comprise the ArcA regulon and the FeHm induced modulation of those genes in NTHi R2866, Hib 10810 and Rd KW20. All three actual genes with decreased expression in a Δ*arcA *mutant are also upregulated during FeHm limitation, in the isolates we have examined (HI0592 is an artifact in the original Rd KW20 annotation and removed in subsequent reannotations [see RefSeq file NC_000907]). Similarly, comparison of the set of genes determined to have increased expression in the Δ*arcA *strain with the FeHm regulation data indicates that of the 19 genes upregulated in Δ*arcA*, 14 show upregulation during FeHm supplementation in at least one of the isolates studied. The data in Table 4 indicates that Hib 10810 and NTHi R2866 show greater similarity with the ArcA regulon than does strain Rd KW20. This may result from the experimental conditions used in the microarrays or may reflect a true difference in the regulation. Since the Arc regulator responds to changes in redox potential perceived as oxidation and/or reduction of membrane bound quinones [47,48], as opposed to oxygen levels *per se*, it is possible that the addition of an excess of exogenous iron may lead to trans-membrane redox alteration via a localized Fenton reaction analagous to that proposed by Liu *et al *[49] resulting in changes in transcription of genes in the ArcA regulon. Previously it has been shown that Δ*arcA *mutants of *H. influenzae *show decreased lethality in a mouse model as well as increased sensitivity to killing by human serum [50]. However, the potential variable regulation observed between the isolates in this study is a clear indication that multiple isolates need to be examined before drawing firm conclusions regarding the extent of a regulon or modulon in an organism, and the role of that modulon in virulence.

**Table 4:** ArcA/FeHm regulated operons in *H. influenzae*

		**Fold Change^c^**		
		
**Gene^a^**	**Description^b^**	**NTHi R2866**	**Hib 10810**	**Hi Rd KW20**
**Down regulated Δ*arcA***				

HI0590	*potE*	-12.75	-5.99	-3.20
HI0591	*speF*	-11.40	-13.78	-3.82
HI0592	Conserved hypothetical protein	ns	ns	ns
HI1349	DPS-Ferritin	-4.12	-3.70	-1.54

**Up regulated Δ*arcA***				

HI0007	Formate dehydrogenase beta subunit *fdxH*	7.49	6.00	3.40
HI0008	Formate dehydrogenase gamma subunit *fdxI*	7.47	5.23	3.20
HI0009	FdhE	3.35	3.43	1.88
HI0018	Uracil DNA glycosylase *ung*	ns	ns	ns
HI0026	Lipoate biosynthesis protein A *lipA*	3.19	2.00	ns
HI0174	tRNA methyltransferase	2.36	2.08	1.93
HI0608	Conserved hypothetical protein	ns	-1.54	-1.49
HI0747	NADH dehydrogenase *ndh*	ns	ns	ns
HI0889	Serine methylase *glyA*	1.83	1.68	-1.09
HI0890	Dephospho CoA kinase *coaE*	2.50	3.48	ns
HI1218	L-lactate permease *lldP*	4.67	3.58	ns
HI1444	5,10 methlyenetetrahydrofolate reductase metF	5.19	3.47	ns
HI1661	2-oxoglutarate dehydrogenase E2 *sucB*	1.56	1.17	-1.18
HI1662	2-oxoglutarate dehydrogenase E1 *sucA*	ns	ns	-1.18
HI1727	Argininosuccinate synthetase *argG*	-2.28	ns	ns
HI1728	Conserved hypothetical protein	ns	1.59	ns
HI1730	Conserved hypothetical protein	ns	1.53	ns
HI1731	Conserved hypothetical protein	ns	2.35	ns
HI1739	L-lactate dehydrogenase *lldD*	1.55	1.63	ns

^a. ^Gene locus. For genes with homologues in Rd KW20 the corresponding Rd KW20 gene locus is given.

^b. ^Name and description of the gene, where known, based on annotation

^c. ^Fold change determined from the microarray data. ns, Non significant

## Conclusion

The current investigation was performed to identify the genes preferentially transcribed under FeHm limitation in two clinically relevant isolates of *H. influenzae *and, by comparison with previously published observations, to begin to define the core FeHm modulon for this species. Since our focus at the outset was the elucidation of potential targets mediating iron and/or heme uptake, our experimental conditions were optimized by Q-PCR analysis of known FeHm repressible genes. By inference, these conditions were expected to identify other genes with similar transcriptional kinetics.

This study identified a putative core set of 37 FeHm-ve genes that are similarly regulated in three unrelated *H. influenzae *isolates. This core contains the majority of the known FeHm uptake genes, including the *hgp*s, the *hxuCBA *operon, and the *tbp *operon. All of these are TonB-dependent receptor complexes and the *tonB *operon was also determined to be FeHm-ve in all isolates examined.

Using the criteria of genes determined to be FeHm-ve with similarity to known TonB-dependent proteins two other genes were identified with a potential role in FeHm uptake; HI0113 and HI369. Both of these genes are subjects of ongoing studies in our laboratory

During the design of the experiments detailed in this manuscript, as well as those previously reported from strain Rd KW20 [25], great care was taken to ensure that each isolate was treated identically to minimize environmental influences other than the FeHm status of the media. Nevertheless, there remain potential strain-specific transcriptional differences observed among the three isolates by microarray. While every effort was taken to ensure that the observed effects result solely from the transition of the culture from FeHm restricted to FeHm supplemented conditions, other environmental stimuli cannot be entirely ruled out. It is possible that in our microarray experiments the removal of a large sample from the culture might perturb oxygen levels in the remaining medium. However, identical control experiments in which smaller samples were removed for Q-PCR analysis (0.5 ml, representing 0.4% culture volume) showed similar regulatory profiles of 94% of the core FeHm genes predicted by microarray. This finding indicates that oxygenation effects due to removal of a large sample volume in the microarray experiments are unlikely to account for the apparent effects of FeHm addition on gene regulation. A second potential environmental condition that could lead to transcriptional changes distinct from the response to FeHm addition is an alteration in the levels of other nutrients between the two time points. The two samples used in the microarray are separated by only a 20 minute period and are drawn from the same culture. This removes any potential flask-to-flask differences. In addition, due to the FeHm restriction, the cultures are growing slowly and are at a relatively low cell density and thus unlikely to be significantly depleting available nutrients during the time period between sampling. A further source of experimentally induced variation may be the temporal separation between individual microarray experiments. To overcome this, the control Q-PCR experiments were performed at the same time, with the same batch of media, under identical conditions. Taken together, these considerations suggest that nutrient depletion is unlikely to play a significant role in the observed outcomes of gene expression analyses. While we propose that the 20 minute period between sampling is too short to observe nutrient related changes in gene transcription in the culture, we have not performed experiments to specifically address this issue and are unable to completely discount this possibility.

Additional File 1 only contains genes for which a fold change >1.5 was determined by microarray in at least one isolate. However, the level of expression of the "unregulated", constitutively expressed FeHm genes (not shown in Additional File 1) is unclear. Are these genes expressed at a low constitutive level or at a level more like upregulated genes? In addition, the independent Q-PCR analysis also emphasize the fact that the reporting of "ns" in Additional File 1 for fold change of a gene is not an indication that that gene is not regulated. Thus, further studies are required to define the actual regulatory status of "ns" genes before the gene may be excluded as part of the core FeHm modulon. These missing pieces of information would have a profound effect on our understanding of the system biology of FeHm uptake and utilization as well as cellular metabolism.

Comparisons of the FeHm responsive genes in the three isolates indicate that NTHi R2866 and Hib10810 are more similar to each other than they are to Rd KW20. This may arise from the fact that the two isolates examined in this study are both recently isolated clinical isolates as opposed to Rd KW20 which has undergone multiple passages on artificial media or it may merely reflect the fact that Rd KW20 has a different mode of regulation than the other isolates. To investigate this aspect further, additional microarray analyses are planned with other genome-sequenced *H. influenzae *isolates.

In constrast with other model organisms, relatively few studies have been published in *H. influenzae *that have examined global transcriptional regulatory networks. Comparative genomic analyses have identified putative members of the FNR and CRP regulons [51] and purine, arginine and aromatic amino acid regulons [52]. Knockouts have identified genes whose transcriptional profiles differ upon disruption of a possible regulatory element or co-effector. Included in these studies are the results of disruption of the *tfoX *(*sxy*) gene involved in regulating competence genes and the *cya *gene responsible for production of cAMP [53]. This latter effect would alter regulation patterns of genes within the CRP regulon. Other studies have examined effects of disruption of the *arcA *and *oxyR *regulators [32,33]. Furthermore, transcriptional effects resulting from specific environmental changes have been examined independently [25] or can be derived from the above studies. Thus, we can determine transcription patterns affected by transition between FeHm availability [25], sugar and nucleotide availability [53], and presence of oxidizing agents [33].

As could be expected, the results of our examination of transcriptional patterns altered by FeHm availability possess degrees of overlap with the results from these previous studies. For example, FeHm restriction can be predicted to result in a profound impact on energy generation in the cell. In fact, many genes previously demonstrated to be upregulated during nutrient limitation in a cAMP-dependent manner are also downregulated in this study upon the addition of FeHm. This suggests that the FeHm depleted cultures had elevated cAMP levels. Yet some genes demonstrate the opposite effect. These data show that gene regulatory networks in *H. influenzae *exhibit complexity beyond mere concurrent activation of independent regulons. For example, the *dps *gene (HI1427), encoding the DPS ferritin protein, appears to be part of the ArcA and OxyR regulons as well as responsive to FeHm levels. Transcripts from the operon comprising the ornithine decarboxylase and putrescine transporter (HI0591 and HI0592) and the HI0608 gene encoding a probable transport permease are negatively impacted by disruption of the *arcA *and *cya *genes compared to the wildtype strain and are also downregulated in response to FeHm addition; yet transcripts the gene encoding lactate permease (HI1218) which decrease in the *cya *mutant, increase in the *arcA *mutant and are upregulated in response to FeHm addition. Studies examining targets of particular transcriptional regulators should be performed in parallel with studies examining the effect of a particular stimulating substance(s) as part of a global approach to define gene regulation. However, the lack of sufficient data from *H. influenzae *makes a thorough examination of iron or heme's affect on independent regulons difficult within the scope of this work.

Overall, the data reveal a core FeHm modulon shared by all of the isolates studied. The core modulon includes genes in known regulons shown to have a role in virulence, such as the ArcA regulon [32]. It is clear that the physiological roles of the genes in the putative core FeHm modulon are broad in the scope of function and may encompass most cellular processes including replication, energy metabolism, solute transport, protection against oxidation and biofilm formation. In addition, several new, previously uncharacterized genes have been identified with a possible role in FeHm metabolism. On the basis of our data it would appear that each of the three isolates may have a distinct set of non-core genes that respond to FeHm availability. Many of these genes are specific to individual isolates (see figure 4). While a common mechanism(s) for coordinated regulation of the core modulon across the species can be postulated, the regulation of the species-specific genes and strain-specific genes pose separate questions.

In summary, the major conclusions of this study are the following. First, the culture, microarray, and Q-PCR methodologies that we previously employed for the analysis of the regulation of gene expression by FeHm in the *H. influenzae *laboratory strain Rd KW20 were successfully extended to two recently sequenced clinical isolates, strains 10810 and R2866. Second, characterization of growth in vitro and conditions for reproducible transcriptional regulation by FeHm permitted direct comparison of gene expression among these three strains under identical environmental conditions. Third, among these three *H. influenzae *strains, a core set of genes responsive to environmental FeHm levels had been defined. The rigor and reproducibility of these microarray data were confirmed independently by Q-PCR. Fourth, these studies demonstrate the utility of comparative transcriptional profiling to identify genes shared across the species that are core members of an important modulon. These genes may play a key role in virulence; they are likely expressed during human disease; and thus, may comprise useful targets for potential therapeutic studies. These studies lay the foundation for further investigation of the role played by the FeHm modulon in *H. influenzae *virulence.

## Methods

### Bacterial strains and routine culture conditions

The Hib strain 10810 was isolated from a case of meningitis and its genome has been completely sequenced [28]. Strain 10810 was kindly provided by Drs. Derrick Crook, Derek Hood and Richard Moxon. The NTHi strain R2866 was isolated from the blood of an immunocompetent child with clinical signs of meningitis subsequent to acute OM [29], and its genome has been completely sequenced [54]. Strain R2866 was kindly provided by Dr. Arnold Smith. *H. influenzae *were routinely cultured at 37°C on chocolate II agar with bacitracin (BBL Prepared Media, Becton Dickinson and Co., Sparks, MD.)

### Growth conditions for iron/heme (FeHm) regulated gene expression

Hemin was purchased from Sigma Chemical Co. (St. Louis, MO.) and used to make stock heme solutions as previously described [55] (heme is correctly defined as ferrous PPIX while hemin is ferric PPIX; however for the purposes of this manuscript heme is used as a general term and does not indicate a particular iron valence state). Growth conditions pertaining to the FeHm-regulation window of *H. influenzae *Rd KW20 have been previously defined [25], and were used as the basis to define growth of strains Hib 10810 and NTHi R2866. For both experimental strains various conditions were systematically evaluated to optimize growth characteristics, maintenance of viability consequent to iron and heme starvation and reproducible regulation of gene expression. The following conditions were found to be optimal for our analysis of the regulation of gene transcription by iron and heme. To prepare the primary inocula *H. influenzae *were initially grown in 15 ml conical tubes containing 5 ml of brain heart infusion (BHI) broth (Difco, Detroit, MI) supplemented with 10 μg/ml β-nicotinamide adenine dinucleotide (BHI-NAD) and additionally supplemented with 0.1 μg/ml heme. These broth cultures were grown at 37°C on a rotator for either 2 hours, in the case of Hib 10810, or for 3 hours, in the case of R2866, and were moderately turbid. The shorter primary incubation time for strain 10810 reflected an apparent faster growth rate for this strain, and was used to ensure that both isolates were at a similar growth phase prior to preparing the individual inocula. For the final inoculum, cells were pelleted by centrifugation, washed once in phosphate buffered saline (PBS) containing 0.1% gelatin and the pelleted cells were re-suspended in the same buffer. The suspension was adjusted to an A_605 nm _= 0.50 and then diluted serially in the same buffer to provide an inoculum giving a final concentration of ~2 × 10^7 ^cfu/ml when 5 ml of inoculum was added to 120 ml BHI-NAD broth additionally supplemented as specified. Broth cultures for iron and heme (FeHm) mediated regulation of gene expression were incubated in a rotary shaker at 175 rpm at 37°C, and 50 μl samples were removed at 30 minute intervals for determination of viable counts. For Q-PCR analyses aliquots of 500 μl were removed at specified times and immediately mixed with 1 ml RNAProtect (Qiagen, Valencia, CA) and frozen at -70°C for later RNA preparation. Sixty milliliter samples for microarray studies were taken at 90 and 110 minutes of incubation, immediately mixed with 60 ml RNAProtect and stored frozen at -70°C for later RNA preparation.

### RNA purification

Samples for Q-PCR obtained as described above were thawed, remixed by brief vortexing and incubated at room temperature for 5 minutes prior to purification using the RNeasy mini kit (Qiagen, Valencia, CA). Following purification, the sample was eluted with 40 μl of sterile RNase free water. Residual chromosomal DNA was removed by digestion with amplification grade DNase I (Invitrogen, Carlsbad, CA). The RNA samples were used to prepare cDNA as previously described [56]. Each 20 μl reaction contained 7 μl template RNA, 5.5 mM MgCl_2_, 500 μM each dNTP (dATP, dCTP, dGTP, dTTP), 1 × RT buffer, 80 mU RNase Inhibitor and 25 U MultiScribe Reverse Transcriptase (Applied Biosystems, Foster City, Ca.). The synthesis reaction was incubated at 25°C for 10 minutes followed by a further 30 minutes at 48°C. The reaction was terminated by heating at 95°C for 5 minutes. Prior to analysis, the cDNA was diluted by addition of 180 μl RNase-free water.

Samples for microarray obtained as described above were thawed and the cells collected by centrifugation. Total RNA was isolated using Trizol (Invitrogen) as described by the manufacturer. Residual genomic DNA was removed by treatment with RNase-free DNase (Invitrogen) as directed by the manufacturer and confirmed by Q-PCR analysis. The RNA samples were then subjected to LiCl precipitation as previously described [57] and concentrations determined using a Smartspec3000 (BioRad, Hercules, CA). Finally, to ensure that the RNA was not degraded, samples were resolved by PAGE using precast 6% TBE-Urea gels (Invitrogen). On receipt at Nimblegen, each sample was subjected to in-house quality control prior to processing samples for microarray analysis.

### Quantitative real-time PCR

(Q-PCR) was performed as previously described [56]. Gene-specific oligonucleotide primers were designed using Primer Express 2.0 (Applied Biosystems) (See Additional File 4: Primers used for Q-PCR analysis) and synthesized by Operon Technologies (Huntsville, AL) and were tested to determine amplification specificity, efficiency and for linearity of the amplification with RNA concentration. A typical 25 μl reaction contained 12.5 μl of SYBR Green Master Mix, 250 nM of each primer, and 5 μl of cDNA sample. Quantification reactions for the target transcripts at each timepoint were performed in triplicate and normalized to concurrently run 16s rRNA levels from the same sample. Relative quantification of gene expression was determined using the 2^-ΔΔCt ^method of Livak and Schmittgen where ΔΔC_t _= (C_t, Target _- C_t,16s_)_Timex _- (C_t, Target _- C_t,16s_)_Control _[58].

### Microarray design

A microarray chip containing probes to all the genes of isolates Hib 10810 and NTHi R2866 was designed. Due to the frequency of phase variation in *H. influenzae *and the possibility of sequencing errors, all frameshifted open reading frames were included on the arrays as a complete gene. Primer sets for the array were designed by Nimblegen Systems, Inc. (Madison, WI). Each ORF of the genomes is represented by thirteen longmer expression probes (60 nucleotides each). The probes were screened for uniqueness to minimize cross-hybridization. Each probe was replicated three times on each chip to increase accuracy.

Arrays were manufactured by NimbleGen Systems, Inc. by maskless array synthesis using a digital micromirror array-mediated, parallel synthesis process incorporating 5'-photoprotected phosphoramidites as previously described. [59].

Post scan, the array features within the image file were extracted using NimbleScan v2.1. This program allows the user to combine the microarray image with the corresponding NimbleGen microarray design file, and optionally, with a gene description file to further map the image. The resulting alignment can be visually manipulated for further analysis. The Expression Data was processed using tools available through the Bioconductor project [60]. Data was normalized using quantile normalization [61], and gene calls generated using the Robust multichip average (RMA) algorithm as described [62].

### Microarray data analysis

Technical array replicates (arising from the presence of three duplicate arrays on each slide) were averaged prior to analysis of the three biological replicates of each isolate. The data were log2 transformed and compared between the two conditions by performing individual *t *tests using the TMEV software [63,64]. Genes with a 1.5-fold expression change and *P *≤ 0.05 were considered significant.

### Annotation of *H. influenzae *genome sequences

Annotation of the genomic sequences of strains Hib 10810 and NTHi R2866 was performed in house (data not shown). The annotation of the NTHi R2866 genome was based on comparative analyses between Rd KW20 [40], NTHi 86-028NP [65] and the NTHi R2846 sequence [54]. Genes in R2866 were predicted using the program GLIMMER [66], trained on the codon usage pattern in strain Rd KW20. Predicted amino acid sequences for each called gene were compared between the four strains to determine consensus start sites and to account for frameshifted genes present in each strain. Manual annotation of nonredundant genes was performed by comparison to complete genomic sequences in other bacterial species. Using the sequences of Rd KW20, 86-028NP, R2846 and R2866, the probable ORFs in the Hib 10810 genome were predicted. The annotated NTHi R2866 genome sequence (with corrected frameshifts) is available upon request.

Genes/proteins prefaced with "*probable*" indicate that there is, within the literature, experimental evidence of a function in homologs in other bacteria, or that a function is proposed on the basis of well-characterized sequence motifs. Proteins prefaced with "*putative*" indicate that the encoded protein contains conserved domains that suggest a possible function. Genes listed as "*conserved hypothetical protein*" display significant sequence homology to predicted proteins outside of the Pasteurellaceae but lack well characterized motifs or experimental evidence for function while genes listed as "*hypothetical proteins*" lack significant homology to other predicted proteins.

### Designation of putative operons

Operons and stand-alone genes were determined based on a number of factors. These include gene cluster analysis across species, transcriptional data from previous studies, conformity of orientation, size of intergenic regions and prediction of putative transcriptional terminators using the program Transterm [67].

### Accession number

The microarray data from this study has been deposited with the Gene Expression Omnibus [68] with the accession number GSE11362.

## Abbreviations

Hib: *Haemophilus influenzae *type b; NTHi: Nontypeable *Haemophilus influenzae*; ORF: Open Reading Frame; OM: Otitis media; FeHm: Iron and heme; FeHm-ve: Iron and heme repressible; FeHm+ve: Iron and heme inducible; BHI: Brain Heart Infusion; BHI-NAD: Brain Heart Infusion supplemented with 10 μg/mlβ-nicotinamide adenine dinucleotide; PPIX: Protoporphyrin IX; ROI: Reactive oxygen intermediates; PCR: Polymerase chain reaction; Q-PCR: Quantitative-polymerase chain reaction; PBS: Phosphate buffered saline.

## Authors' contributions

All authors contributed to the design and execution of the experiments detailed. PWW, TWS, TMV performed microarray analysis. TWS performed growth studies. PWW drafted the manuscript. DJM, TWS and TLS revised the manuscript. All authors read and approved the final manuscript.

## Supplementary Material

Additional file 1 **Comparison of genes identified as FeHm regulated in isolates NTHi R2866, Hib 10810 and Rd KW20.** The data compares fold transcriptional change of genes in three strains of *H. influenzae *in response to iron and heme supplementation of the growth media. The genes shown are only those that exhibit a significant change in the level of transcription in at least one of the three strains. Click here for file

Additional file 2 **Fold transcriptional change of NTHi R2866 genes following supplementation of FeHm-restricted media with exogenous FeHm.** The data represent the transcriptional change of all genes in nontypeable *H. influenzae *strain R2866 in response to iron and heme supplementation of the growth media. Click here for file

Additional file 3 **Fold transcriptional change of Hib 10810 genes following supplementation of FeHm-restricted media with exogenous FeHm**. The data represent the transcriptional change of all genes in type b *H. influenzae *strain 10810 in response to iron and heme supplementation of the growth media. Click here for file

Additional file 4 **Primers used for Q-PCR analysis.** This table lists all primers used in the current study. Click here for file
