# Supplementary material for: The iron/heme regulated genes of Haemophilus influenzae: comparative transcriptional profiling as a tool to define the species core modulon
Source: BMC Genomics. 2009 Jan 7;10:6. doi: 10.1186/1471-2164-10-6 (PMC2627913; doi:10.1186/1471-2164-10-6)
Supplement: Additional file 4 — Primers used for Q-PCR analysis. This table lists all primers used in the current study. [file 1471-2164-10-6-S4.doc]

| **Additional File 4 – Primers used for Q-PCR analysis** | | | | | |
| --- | --- | --- | --- | --- | --- |
| **Primer Name** | **Target Gene** | | **Target strain** | | **Sequence** |
| QPCR-16S-F | 16s rRNA | Rd/ R2866/10810 | | 5’-TCGTCAGCAAGAAAGCAAGCT | |
| QPCR-16S-R | 16s rRNA | Rd/ R2866/10810 | | 5’-GCTGGCGGCAGGCTTAA | |
| QPCR-HI1264-uF | *gyrA* | Rd/ R2866/10810 | | 5’-CGTAAAATCAGCGCGTGTTG | |
| QPCR-HI1264-R1 | *gyrA* | Rd/ R2866/10810 | | 5’-GAGAAGGGTTGTGCCATACGA | |
| QPCR-HI0006m-F1 | *fdxG* | Rd/ R2866/10810 | | 5’-TGAATCCCGTAACACCTGTACATATT | |
| QPCR-HI0006m-uR | *fdxG* | Rd/ R2866/10810 | | 5’-TTGTGCCAGTATGGCTGCTTA | |
| QPCR-HI0007-uF | *fdxH* | Rd/ R2866/10810 | | 5’-GATCGTTTAGAATGGCTGATTCG | |
| QPCR-HI0007-uR | *fdxH* | Rd/ R2866/10810 | | 5’-TGCAGGACAAGCCTTTAAACAA | |
| QPCR-HI0017-F1 |  | Rd/R2866/10810 | | 5’-CTCAAGCGGCAAATGACAATT | |
| QPCR-HI0017-uR |  | Rd/R2866/10810 | | 5’-TCGCCTTTTGCACATAAGCA | |
| QPCR-HI0020-uF |  | Rd/R2866/10810 | | 5’-GCCAGCAACGCCCTCTAA | |
| QPCR-HI0020-uR |  | Rd/R2866/10810 | | 5’-CCACTACGCTATTAATGATTGGAAAGA | |
| QPCR-HI0035-F1 |  | Rd/R2866/10810 | | 5’- GGGCAGGGCAGCAAATT | |
| QPCR-HI0035-uR |  | Rd/R2866/10810 | | 5’- GCGGATTAACCACATTGCAA | |
| QPCR-HI0075-uF | *nrdD* | Rd/R2866/10810 | | 5’-TGGGCAAACACCATTTGTGA | |
| QPCR-HI0075-R1 | *nrdD* | Rd/R2866 | | 5’- TTGCTGAATTAAGCGGGATTG | |
| QPCR-HI0075-R2 |  | 10810 | | 5’- TTGCTGAATTAAACGGGATTG | |
| QPCR-HI0092-uF |  | Rd/R2866/10810 | | 5’- GTGCTTGAAACGCAAAATTTACC | |
| QPCR-HI0092-uR |  | Rd/R2866/10810 | | 5’- GGACGAAGTGCCAATAGCAAA | |
| QPCR-HI0095-uF |  | Rd/R2866/10810 | | 5’- TTGCGTCCAGGCGGTAA | |
| QPCR-HI0095-R1 |  | Rd/R2866/10810 | | 5’- ACCTCTAACACTTTTTTATCTTGGCTAAA | |
| QPCR-HI0097-uF | *hitA* | Rd/R2866/10810 | | 5’-CAGGGCTTTTAGCACCAATTTC | |
| QPCR-HI0097-uR | *hitA* | Rd/R2866/10810 | | 5’-ACGGCCACTTAATGCAATCC | |
| QPCR-HI0098-3-uF | *hitB* | Rd/R2866/10810 | | 5’-TTGCCAATGGCACAAACTACA | |
| QPCR-HI0098-3-uR | *hitB* | Rd/R2866/10810 | | 5’-CCCAAGACTTTGTCCGACTTTTT | |
| QPCR-HI0099-uF | *hitC* | Rd/R2866/10810 | | 5’-TGGCGAAATTTGGCTAAAAGA | |
| QPCR-HI0099-uR | *hitC* | Rd/R2866/10810 | | 5’-GAAGATTAAAATTCTCGCCAAAAATTA | |
| QPCR-HI0113-uF | *tdhA* | Rd/R2866/10810 | | 5’-TGTAACCGCAAAACAAAATTCTTC | |
| QPCR-HI0113-uR | *tdhA* | Rd/R2866/10810 | | 5’-TGTTTTAATACTGAACGACCAGGTAGTTTAT | |
| QPCR-HI0139-uF | *ompP2* | Rd/R2866/10810 | | 5’-TCGCAGCTTCAGCAGCAA | |
| QPCR-HI0139-R1 | *ompP2* | Rd/ 10810 | | 5’-GCTTATTACTTTGTTCTGCGATAATGC | |
| QPCR-HI0139-R2 | *ompP2* | R2866 | | 5’-GTTATTACTTTGTTCTGCGATAACGC | |
| QPCR-HI0152-uF |  | Rd/R2866/10810 | | 5’- CCTTACCCGATGAACTGATTCC | |
| QPCR-HI0152-uR |  | Rd/R2866/10810 | | 5’- ACGACACTGATGACGCTGAAAA | |
| QPCR-HI0153m-uF | *dcuB* | Rd/R2866/10810 | | 5’- TTGCGCATCCGTACTTGAAG | |
| QPCR-HI0153m-uR | *dcuB* | Rd/R2866/10810 | | 5’- CGGAGAATGCGTTCAGCAAT | |
| QPCR-HI0157-uF | *fabH* | Rd/R2866/10810 | | 5’-TGCCAAGTGCAAGGTTTATTAAATAT | |
| QPCR-HI0157-uR | *fabH* | Rd/R2866/10810 | | 5’-CATAGACAAAGCCTGTGCAAGCT | |
| QPCR-HI0164-uF | *nqrA* | Rd/R2866/10810 | | 5’- GCGGGAAAACCAGCACAA | |
| QPCR-HI0164-uR | *nqrA* | Rd/R2866/10810 | | 5’- CATCCCCACATACTCCTCACCTA | |
| QPCR-HI0173-uF |  | Rd/R2866/10810 | | 5’- GCAGCTGCGGTGGTTTATCT | |
| QPCR-HI0173-uR |  | Rd/R2866/10810 | | 5’- AGCGTGTCGCAAGGTTTATC | |
| QPCR-HI0185-F1 | *adhC* | Rd | | 5’-CAGCTGAATGTGGCGAATGT | |
| QPCR-HI0185-F2 | *adhC* | R2866/10810 | | 5’- CTGCTGAATGTGGCGAATGT | |
| QPCR-HI0185-R1 | *adhC* | Rd/10810 | | 5’-CGTGCAATCAGGCATTAAGC | |
| QPCR-HI0185-R2 | *adhC* | R2866 | | 5’- CGTGCAATCAGGCATGAAGC | |
| QPCR-HI0206-uF | *nucA* | Rd/R2866/10810 | | 5’- TGCCAAAGAGGCACCACAA | |
| QPCR-HI0206-uR | *nucA* | Rd/R2866/10810 | | 5’- CGTGCGGTTCTAAATAAGAATGG | |
| QPCR-HI0223-uF |  | Rd/R2866/10810 | | 5’- TATTTATTTAGTATATGGCTCCAACCACTTAG | |
| QPCR-HI0223-uR |  | Rd/R2866/10810 | | 5’- ACCCAAAGATCATGGTCAGCAT | |
| QPCR-HI0230-uF |  | Rd/R2866/10810 | | 5’- CCCACCTTAATCGTGGTTTAAATT | |
| QPCR-HI0230-uR |  | Rd/R2866/10810 | | 5’- TTATCGGCTTGGTAGAACTGTAAGAAAT | |
| QPCR-HI0244-F1 | *tgt* | Rd/R2866/10810 | | 5’- TTACCCAGCGACTTTCGATTATG | |
| QPCR-HI0244-R1 | *tgt* | Rd/R2866/10810 | | 5’- CGCGGCTGCGTTTTG | |
| QPCR-HI0246-F1 |  | Rd/R2866 | | 5’- AACAAACGGTGACTGCTGTGTATC | |
| QPCR-HI0246-F2 |  | 10810 | | 5’- AACAAACTGTGACTGCAGTGTATC | |
| QPCR-HI0246-uR |  | Rd/R2866/10810 | | 5’- TGCCATCGCCCACACTTAC | |
| QPCR-HI0251-uF | *tonB* | Rd/R2866/10810 | | 5’-TCGCTATTGGGTTTGCTTATTTC | |
| QPCR-HI0251-R1 | *tonB* | Rd | | 5’-TGTGCACTATTTGCGCTATCACT | |
| QPCR-HI0251-R2 | *tonB* | R2866/10810 | | 5’-TGTGCGCTATTTGCACTATCACT | |
| QPCR-HI0252-uF | *exbD* | Rd/R2866/10810 | | 5’-GCTCAATGGAATAAAGATCAGAAAGTAA | |
| QPCR-HI0252-uR | *exbD* | Rd/R2866/10810 | | 5’-AAAGCATATCTGTAATCGTCACAAAATC | |
| QPCR-HI0254-uF | *bcp* | Rd/R2866/10810 | | 5’- CACAAGCCTGCGGATTGC | |
| QPCR-HI0254-uR | *bcp* | Rd/R2866/10810 | | 5’- GGGCATCAGGACTAATACCAAGTAC | |
| QPCR-HI0257-F1 |  | Rd/R2866 | | 5’- GGACATCACTCCTGCAATTCG | |
| QPCR-HI0257-F2 |  | 10810 | | 5’- GGACATCACCCCTGCAATTCG | |
| QPCR-HI0257-uR |  | Rd/R2866/10810 | | 5’- TTAACTGCGTTTGCCACTTACCTA | |
| QPCR-HI0262-uF | *hxuC* | Rd/R2866/10810 | | 5’-CGAGGGTTAAGTGATAATCGTGTT | |
| QPCR-HI0262-uR | *hxuC* | Rd/R2866/10810 | | 5’-AGCTACTTGGTCCTTTGATTACTTCAAT | |
| QPCR-HI0263-uF | *hxuB* | Rd/R2866/10810 | | 5’-ACCTCGTTATTCTGTTATTGCAAGTG | |
| QPCR-HI0263-R1 | *hxuB* | Rd/R2866/10810 | | 5’-CACGGTTCAATGATCCAGTATCTG | |
| QPCR-HI0298-uF | *pilB* | Rd/R2866/10810 | | 5’- CCCATTTTGAACAAGAAGATGATG | |
| QPCR-HI0298-R1 | *pilB* | Rd/10810 | | 5’- GGCATTTTTTTGTAAGGCAGATTC | |
| QPCR-HI0298-R2 | *pilB* | R2866 | | 5’- GGCATTTTTTTGTAAGGCGGATTC | |
| QPCR-HI0319-uF | *yecO* | Rd/R2866/10810 | | 5’-TATTTTCTACCCCCATTGCTAAATTG | |
| QPCR-HI0319-uR | *yecO* | Rd/R2866/10810 | | 5’-ATCTGGAAAGACTTCAGCAACGT | |
| QPCR-HI0324-uF | *rnt* | Rd/R2866/10810 | | 5’-TGGCTATTTCCCCGTCATTATT | |
| QPCR-HI0324-uR | *rnt* | Rd/R2866/10810 | | 5’-TGATGGCGGCTAGTTCGAGTA | |
| QPCR-HI0342-uF | *napF* | Rd/R2866/10810 | | 5’-TGCGATTCGTTTCAAATTACAAA | |
| QPCR-HI0342-uR | *napF* | Rd/R2866/10810 | | 5’-AAGCCCCACAGCCATTACAA | |
| QPCR-HI0361-uF | *yfeB* | Rd/R2866/10810 | | 5’-GGCAAGTCCACATTATTTAAAAGCAT | |
| QPCR-HI0361-uR | *yfeB* | Rd/R2866/10810 | | 5’-TGGGAAATTGGCAAATCACAA | |
| QPCR-HI0362-uF | *yfeA* | Rd/R2866/10810 | | 5’-AATGCCGCTGACTATGCTCAA | |
| QPCR-HI0362-R1 | *yfeA* | Rd/R2866 | | 5’-GCGCTTCTGGAATTTGTGCTA | |
| QPCR-HI0362-R2 | *yfeA* | 10810 | | 5’-GCGCTTCTGGAATCTGTGCTA | |
| QPCR-HI0365-uF |  | Rd/R2866/10810 | | 5’-GGATTTAACGCGCCAACAA | |
| QPCR-HI0365-uR |  | Rd/R2866/10810 | | 5’-TCTTCGCCAAAATGATAAATCCA | |
| QPCR-HI0502-uF | *rbsA* | R2866/10810 | | 5’- TCGATCAGTTAATTTCAATGGACCTA | |
| QPCR-HI0502-uR | *rbsA* | R2866/10810 | | 5’- AGATTACCGACCAAATTCAGTTCTTG | |
| QPCR-HI0507-uF |  | Rd/R2866/10810 | | 5’- AACCTGTTGATCCTTATGCGAAATA | |
| QPCR-HI0507-uR |  | Rd/R2866/10810 | | 5’- GCAACCAGCGACTAGCAAAAAT | |
| QPCR-HI0534-uF | *aspA* | Rd/R2866/10810 | | 5’- AAGTGATATTGCAAAAGCGATTGTA | |
| QPCR-HI0534-R1 | *aspA* | Rd/R2866/10810 | | 5’- ACGTCTGATGGGAATTGATCTAAGC | |
| QPCR-HI0563-uF | *asnC* | Rd/R2866/10810 | | 5’- GCGAGAACACCATACGCAGAA | |
| QPCR-HI0563-uR | *asnC* | Rd/R2866/10810 | | 5’- TTTTCGACCCGAACGTGAA | |
| QPCR-HI0584-uF | *ilvH* | Rd/R2866/10810 | | 5’- ACGATGGTTTTATGCATGCTTGT | |
| QPCR-HI0584-uR | *ilvH* | Rd/R2866/10810 | | 5’- GCGATCCATAATGCAACACCTAA | |
| QPCR-HI0591-uF | *speF* | Rd/R2866/10810 | | 5’- CTTTTGACTGTCCGGGACATC | |
| QPCR-HI0591-uR | *speF* | Rd/R2866/10810 | | 5’- ACCGTAGAAGTCATAAAGGAAACGA | |
| QPCR-HI0601-F | *tfoX* | Rd/R2866/10810 | | 5’-GCTTTTGGCGAGGATTGGAT | |
| QPCR-HI0601-R | *tfoX* | Rd/R2866/10810 | | 5’-TCAGCTAAAGCAACCGAAACC | |
| QPCR-HI0623-uF | *fmt* | Rd/R2866/10810 | | 5’- GGTTGTTTGAATGTGCATGGTT | |
| QPCR-HI0623-uR | *fmt* | Rd/R2866/10810 | | 5’- GCCCAAATTGAACGCTGAA | |
| QPCR-HI0661-uF | *hgpB* | Rd/R2866/10810 | | 5’-CTGTTGTTGAAGCTGGACGTTTT | |
| QPCR-HI0661-uR | *hgpB* | Rd/R2866/10810 | | 5’-TTCAGCTTGACGTAATCCATCAAT | |
| QPCR-HI0663m-uF |  | Rd/R2866/10810 | | 5’- GAGTTCTTTATTCCGCTTCGTTTG | |
| QPCR-HI0663m-uR |  | Rd/R2866/10810 | | 5’- TCTTATCTGAAGCCGCTTTGC | |
| QPCR-HI0669-uF | *mioC* | Rd/R2866/10810 | | 5’- TGCCGAGCATTTAAACGATGT | |
| QPCR-HI0669-uR | *mioC* | Rd/R2866/10810 | | 5’- TCAGATAAATTTGGTCCGTGAAAA | |
| QPCR-HI0670-uF |  | Rd/R2866/10810 | | 5’- GCCCTAATTCAGCGTGTGTC | |
| QPCR-HI0670-uR |  | Rd/R2866/10810 | | 5’- ACCTTTGCCAATTTTCCCTATTG | |
| QPCR-HI0682-F1 | *ilvC* | Rd/R2866/10810 | | 5’- AGCATGGGCTGCTGCAA | |
| QPCR-HI0682-uR | *ilvC* | Rd/R2866/10810 | | 5’- TCAGATTTTACTTCTGCCACGAAT | |
| QPCR-HI0689-uF | *glpQ* | Rd/R2866/10810 | | 5’- AGAAGTGGTTAAATATGCCGATGGT | |
| QPCR-HI0689-uR | *glpQ* | Rd/R2866/10810 | | 5’- CACAATATTATCAGGTTTGGATTCTTCTT | |
| QPCR-HI0691-uF | *glpK* | Rd/R2866/10810 | | 5’- ACGCGATCGTTTGGCAAT | |
| QPCR-HI0691-uR | *glpK* | Rd/R2866/10810 | | 5’- TGGCCATCGGCTTTTAATTT | |
| QPCR-HI0752-uF | *purL* | Rd/R2866/10810 | | 5’-AATGGTGGCCGTCAAGCA | |
| QPCR-HI0752-uR | *purL* | Rd/R2866/10810 | | 5’-GCAAACATATAAAGCTCCACATCCT | |
| QPCR-HI0774-uF | *atoD* | Rd/R2866/10810 | | 5’-AAACTGTCATTGCCCAAGTTGA | |
| QPCR-HI0774-uR | *atoD* | Rd/R2866/10810 | | 5’-ATGCGGCTGGCGTGAT | |
| QPCR-HI0809-uF | *pckA* | Rd/R2866/10810 | | 5’-TGGTACGGCGGCGAAAT | |
| QPCR-HI0809-uR | *pckA* | Rd/R2866/10810 | | 5’-GCAGTGCATTGCACCAACA | |
| QPCR-HI0811-uF | *argH* | Rd/R2866/10810 | | 5’-CACTTAGTTCAAACCGCAGAAAAC | |
| QPCR-HI0811-uR | *argH* | Rd/R2866/10810 | | 5’-TGGTTGAGCTCGTTGTAAATGG | |
| QPCR-HI0833-uF | *frdC* | Rd/R2866/10810 | | 5’-CGTAAAAAATATGTTCGTCCAATGAC | |
| QPCR-HI0833-uR | *frdC* | Rd/R2866/10810 | | 5’-AGTCCAATTTTTGCCACCAAGT | |
| QPCR-HI0845-uF |  | Rd/R2866/10810 | | 5’-CTCGGATTTGAGTTTAATGGAAATTT | |
| QPCR-HI0845-uR |  | Rd/R2866/10810 | | 5’-GCTCATATTTATCTGTACCATCCATTTTT | |
| QPCR-HI0863-uF | *pdxH* | Rd/R2866/10810 | | 5’-CGCGCTGACTTTCTTTTGG | |
| QPCR-HI0863-uR | *pdxH* | Rd/R2866/10810 | | 5’-TGCTGGAATTTTAACCGCCTTA | |
| QPCR-HI0864-F1 |  | Rd/R2866 | | 5’- CGTATTAAGCGCGGTTCGAT | |
| QPCR-HI0864-F2 |  | 10810 | | 5’- CGTATTAAGCGTGGTTCGAT | |
| QPCR-HI0864-uR |  | Rd/R2866/10810 | | 5’- CGTTGTAAACCAAGATGTCCAAGTAC | |
| QPCR-HI0878-uF |  | Rd/R2866/10810 | | 5’-AACCGTCAGTTGCACAAATTTTT | |
| QPCR-HI0878-uR |  | Rd/R2866/10810 | | 5’-TTGGTGCAACCCTAATTTTTCTTT | |
| QPCR-HI0889-uF | *glyA* | Rd/R2866/10810 | | 5’-AAGATGAAAATCGTCGTCAAGAAGA | |
| QPCR-HI0889-R1 | *glyA* | Rd/R2866/10810 | | 5’-TGTGAACCTTGCGCTTCCA | |
| QPCR-HI0890m-uF | *coaE* | Rd/R2866/10810 | | 5’-GATCTTGGCGTACCTCTAGTGGAT | |
| QPCR-HI0890m-uR | *coaE* | Rd/R2866/10810 | | 5’-GACGACTTCTCTAGCAACCACATC | |
| QPCR-HI0980-uF | *fis* | R2866/10810 | | 5’- TCAGTATTAAATGCGCAATCACAA | |
| QPCR-HI0980-R1 | *fis* | R2866 | | 5’- AATAATTACGCAATGCTTGCTTGA | |
| QPCR-HI0980-R2 | *fis* | Rd/10810 | | 5’- AATAATTACGCAACGCTTGTTTGA | |
| QPCR-HI0994-uF | *tbp1* | Rd/R2866/10810 | | 5’-AAGTAAGAGATCGTAAAGATAATGAAGTAACTG | |
| QPCR-HI0994-uR | *tbp1* | Rd/R2866/10810 | | 5’-ACCGCGACCTTGTTCTACAACT | |
| QPCR-HI0997m-F1 |  | R2866 | | 5’- AACGAATATAGCTTGGGCAGAAGTT | |
| QPCR-HI0997m-F2 |  | 10810 | | 5’- AACCAATGTAGCTTGGGCAAAAGTT | |
| QPCR-HI0997m-F4 |  | Rd | | 5’-AACGAATGTAGCTTGGGCAGAAGTT | |
| QPCR-HI0997m-uR |  | R2866/10810 | | 5’- GAGGTTTTTAATTCCGCACTTTGA | |
| QPCR-HI0999-uF | *rnpA* | Rd/R2866/10810 | | 5’- TCGAACAGCCATTCAGAGCTAGT | |
| QPCR-HI0999-uR | *rnpA* | Rd/R2866/10810 | | 5’- CCTAAACGCGGATGTTCAAGA | |
| QPCR-HI1010-uF |  | Rd/R2866/10810 | | 5’- TGTTATGGCATCGGGTTCAA | |
| QPCR-HI1010-uR |  | Rd/R2866/10810 | | 5’- TGCCGCAGTGGCATCTAA | |
| QPCR-HI1047-uF | *dmsA* | Rd/R2866/10810 | | 5’-AACTGTGGTAGCCGTTGTCCAT | |
| QPCR-HI1047-uR | *dmsA* | Rd/R2866/10810 | | 5’-CCCCGTATTATCGGTTTCCA | |
| QPCR-HI1051-uF |  | Rd/R2866/10810 | | 5’-TCGTTTAATGCTTGGACAAAGTTT | |
| QPCR-HI1051-uR |  | Rd/R2866/10810 | | 5’-CAAGGGCGGTTTGCATTACT | |
| QPCR-HI1064m-uF |  | Rd/R2866/10810 | | 5’-GCGGTGAAATTTGGCATAAAA | |
| QPCR-HI1064m-R1 |  | Rd/R2866/10810 | | 5’-CATGTTGTAAGCAAAAAGCAATGTC | |
| QPCR-HI1069-uF | *nrfA* | Rd/R2866/10810 | | 5’-TGCAAATGATGGCCCTCAA | |
| QPCR-HI1069-uR | *nrfA* | Rd/R2866/10810 | | 5’-GCGATTAAACGTGGAACATCAG | |
| QPCR-HI1094-uF | *ccmF* | Rd/R2866/10810 | | 5’-GGGATCCAGTAGAAAACTCATCGT | |
| QPCR-HI1094-uR | *ccmF* | Rd/R2866/10810 | | 5’-CTGTCACAGAAAGAGAGTGAATCAATG | |
| QPCR-HI1107-uF | *nhaC* | Rd/R2866/10810 | | 5’-ATCGCACAAGAATGCCAACA | |
| QPCR-HI1107-uR | *nhaC* | Rd/R2866/10810 | | 5’-ACCCAAGCCCTAACAGTAATAGCA | |
| QPCR-HI1173-F1 | *sprT* | Rd/R2866 | | 5’-GCAGGCGTGGCTTACCTACA | |
| QPCR-HI1173-F2 | *sprT* | 10810 | | 5’-AGCTCATGTGGCACGACTTG | |
| QPCR-HI1173-R1 | *sprT* | Rd/R2866 | | 5’-GCAGGCGTGGCTTACCTGCA | |
| QPCR-HI1173-R2 | *sprT* | 10810 | | 5’-AGCTCGTGTGGCACGACTTG | |
| QPCR-HI1174m-uF |  | Rd/R2866/10810 | | 5’-ACCAATCGCGCAGAACAAA | |
| QPCR-HI1174m-uR |  | Rd/R2866/10810 | | 5’-TGCTACAACCCCATATCCGAG | |
| QPCR-HI1180-uF | *artP* | Rd/R2866/10810 | | 5’- AATGGCAAATCCCAAAGCAA | |
| QPCR-HI1180-uR | *artP* | Rd/R2866/10810 | | 5’- CGGCCAAAGATGATATTGTTGA | |
| QPCR-HI1190-uF |  | Rd/R2866/10810 | | 5’- CCGATGGATCACGCCTTT | |
| QPCR-HI1190-uR |  | Rd/R2866/10810 | | 5’- TTGCAATAGAGTGGCAATTTGG | |
| QPCR-HI1210-F1 | *mdh* | Rd/R2866/10810 | | 5’-AAGTTGCTGTATTAGGTGCCGC | |
| QPCR-HI1210-uR | *mdh* | Rd/R2866/10810 | | 5’-AATAACGCTAATGCTTGACCAATACC | |
| QPCR-HI1214-uF | *recJ* | Rd/R2866/10810 | | 5’- TGAAAGAAAAAGGCATTCGAGTTT | |
| QPCR-HI1214-uR | *recJ* | Rd/R2866/10810 | | 5’- GGCTGGCGGCAAAGTTT | |
| QPCR-HI1217-F1 | *hup* | Rd | | 5’-ACTTGGCCTAATTAATACGATCGGTAT | |
| QPCR-HI1217-F2 | *hup* | R2866/10810 | | 5’-ACTTGGCCTAATTAATACAATCGGTAT | |
| QPCR-HI1217-uR | *hup* | Rd/R2866/10810 | | 5’-TTTCCACTACATCAATTTGTCCTAATG | |
| QPCR-HI1218-uF | *lctP* | Rd/R2866/10810 | | 5’- AAATATTATGCGAAAATGGCTAGGA | |
| QPCR-HI1218-R1 | *lctP* | Rd/10810 | | 5’- AAGCAAACGCCCAACCAAT | |
| QPCR-HI1218-R2 | *lctP* | R2866 | | 5’- AGGCAAACGCCCAACCAAT | |
| QPCR-HI1224-uF | *pyrF* | Rd/R2866/10810 | | 5’- CAAATACGGTAGCAAGAGCAGTTC | |
| QPCR-HI1224-uR | *pyrF* | Rd/R2866/10810 | | 5’- CACTTGCGTGTAAATCCACCAT | |
| QPCR-HI1228-uF | *upp* | Rd/R2866/10810 | | 5’- CGTGAAGCTGAGATTGATACAAAAA | |
| QPCR-HI1228-uR | *upp* | Rd/R2866/10810 | | 5’- TAAATCAGAGGTTGCTTCATAGGTTAATAG | |
| QPCR-HI1275-uF | *tehB* | Rd/R2866/10810 | | 5’-CTGCAAAAATTATCTCGCCTTGT | |
| QPCR-HI1275-uR | *tehB* | Rd/R2866/10810 | | 5’-CCAAGAGGTCACATCATAGCCTAATA | |
| QPCR-HI1282-uF |  | Rd/R2866/10810 | | 5’- CGCAATTTGAACGCTATATTGG | |
| QPCR-HI1282-uR |  | Rd/R2866/10810 | | 5’-GCCATTTACGGCGTTCCAT | |
| QPCR-HI1305-uF | *thiL* | Rd/R2866/10810 | | 5’-GTCTATTACGATTACGGCACAAGGT | |
| QPCR-HI1305-uR | *thiL* | Rd/R2866/10810 | | 5’- TGTGCTTTATGCCGACAAATTC | |
| QPCR-HI1349-uF | *dps* | Rd/R2866/10810 | | 5’- TGTCAAAAACATCAATCGGACTAGA | |
| QPCR-HI1349-uR | *dps* | Rd/R2866/10810 | | 5’- GGTAGGTTGCAAGTAATTCGTTAAGTT | |
| QPCR-HI1356-uF | *malQ* | Rd/R2866/10810 | | 5’-ATCCTCGAAATGCTTATGCCACTA | |
| QPCR-HI1356-R1 | *malQ* | Rd/10810 | | 5’-TAATTCTAAATCACGACAATGCCAAA | |
| QPCR-HI1356-R2 | *malQ* | R2866 | | 5’-TAATTCTAAATCACGGCAATGCCAAA | |
| QPCR-HI1359-F1 | *glgC* | Rd/R2866/10810 | | 5’- TTGCAAACAGGCTGGTCTTTC | |
| QPCR-HI1359-uR | *glgC* | Rd/R2866/10810 | | 5’- GCTGACGAGCTGGCAACATAT | |
| QPCR-HI1366-uF | *acpD* | Rd/R2866/10810 | | 5’- TGTTTATGCACAAGGCATTGG | |
| QPCR-HI1366-uR | *acpD* | Rd/R2866/10810 | | 5’- CGGACGCTTGGGCTTTT | |
| QPCR-HI1368-F | *pqqL* | Rd/R2866/10810 | | 5’- GAACGTGGCGTAGTACAAGAAGAA | |
| QPCR-HI1368-R1 | *pqqL* | R2866 | | 5’- ATACATAACGAGAACCCGCCATT | |
| QPCR-HI1368-R2 | *pqqL* | Rd/10810 | | 5’- ACACATAACGAGAACCCGCCATT | |
| QPCR-HI1383m-uF | *ptsS* | Rd/R2866/10810 | | 5’- CTTTTGCTCAAGCTAATAGCATCACT | |
| QPCR-HI1383m-uR | *ptsS* | Rd/R2866/10810 | | 5’- TGCCCACTTCGCATAAATTG | |
| QPCR-HI1384-uF | *ftnA* | Rd/R2866/10810 | | 5’-GCTGAACGACCAAATTAACTTAGAGTT | |
| QPCR-HI1384-uR | *ftnA* | Rd/R2866/10810 | | 5’-GCATGACGAAGTAAGAAGGTAGCA | |
| QPCR-HI1398-uF | *fumC* | Rd/R2866/10810 | | 5’- ATGATACTTTCCCAACGGCAAT | |
| QPCR-HI1398-uR | *fumC* | Rd/R2866/10810 | | 5’- TAAACGTTCAACACAAGGAATGGT | |
| QPCR-HI1427-F |  | Rd/R2866/10810 | | 5’- AGAGTTGGTTTACCGCTAGAAATTG | |
| QPCR-HI1427-R |  | Rd/R2866/10810 | | 5’- ACGGTTCGCCTGATTTCG | |
| QPCR-HI1444-uF | *metF* | Rd/R2866/10810 | | 5’- CCGTTCTGTCGCTGATTTTGA | |
| QPCR-HI1444-uR | *metF* | Rd/R2866/10810 | | 5’- TGCTTGTGCGGATTTTGCT | |
| QPCR-HI1445-uF | *msrA* | Rd/R2866/10810 | | 5’- CATTTCTATTTATTACCGCACTTTGC | |
| QPCR-HI1445-R1 | *msrA* | Rd | | 5’- TTTGTATTGCTAAAGTTGGTGTAGCA | |
| QPCR-HI1445-R2 | *msrA* | R2866/10810 | | 5’- TTTGTATTGCTAAAGTTGGTGCTGCA | |
| QPCR-HI1466m-uF |  | Rd/10810 | | 5’-ATCGCAAACCTGAATCACTCATT | |
| QPCR-HI1466m-uR |  | Rd/10810 | | 5’-ATAGCATCAAACATACCACCTAACGT | |
| QPCR-HI1546-uF | *impA* | Rd/R2866/10810 | | 5’-TACTGTATCAAACGTCCAAAACAAACT | |
| QPCR-HI1546-R1 | *impA* | Rd/R2866/10810 | | 5’-CTAACATATCGCCGCTTTCAATT | |
| QPCR-HI1607-uF |  | Rd/R2866/10810 | | 5’- CACTTTTTGCCACTGCGATTC | |
| QPCR-HI1607-uR |  | Rd/R2866/10810 | | 5’- ATTGTACATTAGTAGGACGTTCCATATCTAA | |
| QPCR-HI1612-uF |  | Rd/R2866/10810 | | 5’- GGCATTGCCGCCTACAATT | |
| QPCR-HI1612-uR |  | Rd/R2866/10810 | | 5’- TGCCTTGGCGAACTTGATG | |
| QPCR-HI1693-uF | *modA* | Rd/R2866/10810 | | 5’- AAAAGTGCGGTAAATTCTGTGGAT | |
| QPCR-HI1693-uR | *modA* | Rd/R2866/10810 | | 5’- AGGATCGCCAACAGATAAATAGCT | |
| QPCR-HI1706-F1 | *betT* | Rd | | 5’-CCACATTAGGTTTTGGTTCATCAC | |
| QPCR-HI1706-F2 | *betT* | R2866 | | 5’- CTACATTAGGTTTCGGTTCATCAC | |
| QPCR-HI1706-F3 | *betT* | 10810 | | 5’- CTACATTAGGTTTTGGTTCATCAC | |
| QPCR-HI1706-R1 | *betT* | Rd/10810 | | 5’- ACCACAACAATAACGCCAACTTG | |
| QPCR-HI1706-R2 | *betT* | R2866 | | 5’- ACCACAACAATAACGCTAACTTG | |
| QPCR-HI1733-uF | *rnb* | Rd/R2866/10810 | | 5’-CGTGGTAAAAAGCACAGATAAAGC | |
| QPCR-HI1733-uR | *rnb* | Rd/R2866/10810 | | 5’-ATGGCGGTGCGATGAAATAG | |
| QPCR-HI1738-uF |  | Rd/R2866/10810 | | 5’-ACCCAATTACTGAAGCGGCTAA | |
| QPCR-HI1738-uR |  | Rd/R2866/10810 | | 5’-CCGTATGCTATGCCAAGAAATAAA | |
| QPCR-tnaA-uF | *tnaA* | R2866/10810 | | 5’- TTAAAGATAAATCAATGGAGGAAGTCTATC | |
| QPCR-tnaA-uR | *tnaA* | R2866/10810 | | 5’- CAACACAAAGCGTTCTACATTCGT | |
